# Determinants of Spatial Heterogeneity of Functional Illiteracy among School-Aged Children in the Philippines: An Ecological Study

**DOI:** 10.3390/ijerph16010137

**Published:** 2019-01-07

**Authors:** Kei Owada, Mark Nielsen, Colleen L. Lau, Laith Yakob, Archie C.A. Clements, Lydia Leonardo, Ricardo J. Soares Magalhães

**Affiliations:** 1 School of Medicine, The University of Queensland, South Brisbane, QLD 4101, Australia; 2 Children’s Health and Environment Program, Child Health Research Centre, The University of Queensland, South Brisbane, QLD 4101, Australia; colleen.lau@anu.edu.au (C.L.L.); r.magalhaes@uq.edu.au (R.J.S.M.); 3 Spatial Epidemiology Laboratory, School of Veterinary Science, The University of Queensland, Gatton, QLD 4343, Australia; 4 School of Psychology, The University of Queensland, St Lucia, QLD 4072, Australia; m.nielsen@psy.uq.edu.au; 5 Faculty of Humanities, University of Johannesburg, Auckland Park 2006, South Africa; 6 Research School of Population Health, Australian National University, Canberra, ACT 0200, Australia; archie.clements@anu.edu.au; 7 Department of Disease Control, London School of Hygiene and Tropical Medicine, London WC1E 7HT, UK; laith.yakob@lshtm.ac.uk; 8 Department of Parasitology, College of Public Health, University of the Philippines Manila, Manila 1000, Philippines; lydialeonardo1152@gmail.com

**Keywords:** school-aged children, functional literacy, cognitive function, geographical variation, risk factors, The Philippines

## Abstract

Functional literacy is one of the targets of the Sustainable Development Goals (SDGs) of the United Nations. Functional literacy indicators are likely to vary between locations given the geographical variability of its major determinants. This property poses a challenge to decisions around efficient allocation of population services and resources to mitigate the impact of functional literacy in populations most in need. Using functional literacy indicators of 11,313 school-aged children collected in 2008 during the nationwide survey, the current study examined the association between functional literacy and geographical disparities in socioeconomic status (SES), water supply, sanitation and hygiene, household education stimuli, and environmental variables in all three regions of the Philippines (Luzon, the Visayas, and Mindanao). Three nested fixed-effects multinomial regression models were built to determine associations between functional literacy and a wide array of variables. Our results showed the general prevalence rate of functional illiteracy as being 4.7%, with the highest prevalence rate in the Visayas, followed by Mindanao and Luzon (7.5%, 6.9%, and 3.0%, respectively. Our results indicated that in Luzon prevalence of functional illiteracy was explained by variation in household education stimuli scores, sources of drinking water, and type of toilet facility. In Mindanao and the Visayas prevalence of functional illiteracy was primarily explained by geographical variation in SES, and natural environmental conditions. Our study highlights region-specific determinants of functional literacy and the need for geographically targeted, integrated interventions.

## 1. Introduction

According to the latest United Nations Educational Scientific and Cultural Organization (UNESCO) report on global literacy, there are 114 million illiterate adolescents and youths (15 to 24 year olds) around the world, two-thirds of whom are female [1]. Despite widespread acknowledgement of this problem, between 2000 and 2015, global literacy rates were estimated to have improved by just 4%. Studies have shown that poverty has a considerable impact on psychological functioning and that, for children, this impact increases in severity the longer they live in poverty [2,3,4].

Progress in the Philippines to address national literacy rates has been slow [5]. The literacy rate of individuals aged 15 to 24 years improved from 93.4% in 2003 to 98.1% in 2013 [6,7]. This improvement is likely to be due to increased school opportunities, education expansion, better access to barangay (small administrative divisions in the Philippines) health stations, improvement in general health status, decreased poverty rate, and reduced burden of diseases [8,9]. However, this may also partly be due to measurement errors, and differences in survey designs and tools used by the national statistics office in different years [10]. Despite the overall improvement, there are areas in the Philippines where functional literacy remains low [11].

There are numerous measurement tools available to assess different domains of cognitive function at different stages of childhood [12,13]. One indicator at the population level is functional literacy. Although the meaning of functional literacy varies between countries, regions, and cultures, it is generally understood to be the ability to use a set of cognitive skills to engage in reading, writing, and numeracy for effective functioning and development of individuals and their communities. Conversely, functional illiteracy is therefore defined as the inability to use cognitive skills to engage in reading, writing, and numeracy [14].

Previous studies have demonstrated a clear link between literacy and cognitive abilities [15,16]. Cognitive development of children is influenced by the interplay of several factors. Evidence from longitudinal studies in children indicates that neurocognitive development is impacted by nutritional status, poverty, stress, low maternal education attainment, less nurturing child-rearing environments, younger maternal age, living in a rural environment, and poor psychosocial stimulation at home and at school [17,18,19]. Cognitive processes that result in functional illiteracy can be influenced not only by individual and household factors but also by environmental determinants. Cognitive impairments are multifactorial in nature and both non-communicable (e.g., malnutrition) and communicable diseases (e.g., malaria and neglected tropical diseases) are known contributors [20]. The exposure to many of these conditions is determined by anthropogenic environmental factors (i.e., human impacted habitat conditions) such as sanitation, urbanisation, and overcrowding [21], and natural environmental factors such as elevation, temperature, and water availability [22].

Functional literacy indicators are likely to vary between locations given the geographical variability of its major determinants. This property poses a challenge to decisions around efficient allocation of population services and resources to mitigate the impact of functional literacy in populations most in need. The application of spatial epidemiological approaches has been useful for identifying areas where the risk of diseases is at its highest and for highlighting areas where interventions are most needed. Spatial epidemiological approaches have been utilised to address such challenges globally [22,23,24], and in the Philippines, mainly looking at infectious diseases such as soil-transmitted helminths [25,26,27]. However, at present no study has attempted to investigate the spatial epidemiology of functional illiteracy and quantify the role of different determinants in functional illiteracy. Such a study would provide an evidence base for understanding local burden of functional illiteracy and assist in geographical targeting of interventions.

In this study, we aimed to quantify geographical disparities in functional literacy in the Philippines and the role of socioeconomic status (SES), water supply, sanitation and hygiene (WASH), household education stimuli, and environmental variables in the observed geographical disparities.

## 2. Materials and Methods

### 2.1. Study Area

The Philippines is situated in Southeast Asia in the western Pacific Ocean and consists of 7107 islands in three main geographical regions: Luzon, the Visayas, and Mindanao [7]. The smallest administrative division is the barangay (average diameter of 11 km) of which there are a total of 42,028 [7]. At the last census (2015), the total population was 100.98 million, up from 92.34 million in the previous census (2010), with ~30 million individuals aged 5 to 19 years [6]. Ethical clearance for this analytical study was provided by the University of Queensland School of Medicine Low Risk Ethical Review Committee (clearance number 2014-SOMILRE-0100), and approved on 20 June 2014.

### 2.2. Data for Analysis

#### 2.2.1. The 2008 Functional Literacy, Education and Mass Media Survey (FLEMMS)

The 2008 FLEMMS is the fourth in a series of functional literacy surveys conducted in the Philippines. The previous three rounds were conducted in 1989, 1994, and 2003. The 2008 FLEMMS survey used the 2003 survey master sample (MS) created for household surveys on the basis of the 2000 Census Population and Housing (CPH) results [11]. In brief, for each region, a three-stage sampling scheme was used: the selection of primary sampling units (PSU) for the first stage, of sample enumeration areas (EA) for the second stage, and of sample housing units for the third stage. The FLEMMS sampling scheme is described in detail in Text S1. The heads of households were administered with the household questionnaire, and all information was gathered by trained interviewers (a copy of the FLEMMS forms available for reference from corresponding author). All members 10 to 64 years old in the sampled households, regardless of educational attainment, were provided with a self-administered individual questionnaire (prepared in English and translated into 26 local languages commonly spoken in the selected sample areas) [11]. The design of FLEMMS was verified and the survey was conducted by trained interviewers, with high response rates. Furthermore, data collected from the survey were cleaned and verified by the national statistics office. Additional information on the design of the survey and how the data were processed are detailed in the FLEMMS report [11].

#### 2.2.2. Functional Literacy Data

Functional literacy rates were estimated based on seven themes or questions included in the FLEMMS individual questionnaire, using a value set of 1 ‘satisfactory’ and 2 ‘not satisfactory’ (which also includes ‘not answered independently’ or ‘no answer’). Themes included (1) name; (2) address; (3) date of birth; (4) highest school grade completed; (5) first simple arithmetic questions e.g., if a kilo of rice costs P25.00, how much will two kilos cost?; (6) second simple arithmetic questions e.g., if a kilo of sugar costs P38.00, how much will half a kilo cost?; and (7) reading comprehension [11]. All items were categorised into dichotomous variables (satisfactory or not satisfactory). A summary of the individual scores was used to determine the functional literacy level. Reading and writing skills were assessed based on the scores of themes 1 to 4, arithmetic skills on themes 5 and 6, and comprehension skills on theme 7.

Our study focuses on school-aged population. For the purpose of our analysis, school-aged children were defined as 10 to 19 years old (school-aged children and adolescents). FLEMMS survey did not collect information on children under 10 years old. In total, we analysed data from 11,313 school-aged children with complete information regarding functional literacy and demographic variables. Household level variables (e.g., socioeconomic status, WASH variables), and information regarding head of households such as adult functional literacy and education attainment were measured for a total of 10,339 heads of households which was extracted from FLEMMS household questionnaire (Text S2).

A total of 1506 barangays were included, 851 located in Luzon, 254 in the Visayas, and 401 in Mindanao (Figure S1).

#### 2.2.3. Sociodemographic Indicators

We used data from the FLEMMS household and individual questionnaires on school-aged children and the heads of households, including age, sex, marital status, education level, employment, and occupation status. This indicator is described in detail in Text S2.

#### 2.2.4. Water Supply, Sanitation and Hygiene (WASH) Indicators

We used data from the FLEMMS household questionnaire on the following WASH indicators: (a) main sources of drinking water (piped into dwelling, protected well, unprotected well, lake or pond or rain water or rivers, and other); (b) the types of toilet facility at home (flush toilet, pit toilet, and no toilet or bush or field); (c) main material of floor (natural floor earth or sand, bamboo or palm or wood, cement, and other); (d) main material of roof (palm or bamboo or wood, aluminium, and other); and (e) main material of outer walls of houses (bamboo or cane or palm or wood, cement, and other).

#### 2.2.5. Socioeconomic Status (SES) Indicators

We used data from the FLEMMS household questionnaire which included a poverty indicator (dichotomous variable: poor or non-poor) generated using ownership of household amenities and conveniences (e.g., whether the home had electricity, refrigerator, washing machine, phone, cell phones, TV, CD, Karaoke or video machine, personal computer, tractor, boat, car, tricycle, motorcycle, bicycle) [11]. We used this binary poverty indicator as a proxy of household-level SES in order to classify households as either low or high SES.

#### 2.2.6. Household Education Stimuli and Cognitive Stimulation

A total of 19 close-ended questions were selected from FLEMMS household questionnaire as the home inventory-proxy items. These included questions such as ‘Does your family read newspapers?’, ‘Does your family listen to radio?’, and ‘Is there a personal computer at home?’ The items were used to construct cognitive stimulation sub-indices. All of the individual items were translated into dichotomous (yes or no) variables. The total score is the summation of the individual item scores and was used as a covariate in our models. The components of the sub-indices are specified in Text S2 and Table S1.

#### 2.2.7. Environmental Data

Data for average annual land surface temperature (LST), average annual rainfall, and distance to perennial water bodies (DPWB) were obtained from WorldClim (www.worldclim.org). The normalised difference vegetation index (NDVI), which serves as a proxy measure of rainfall for a 1 km × 1 km grid cell resolution, was obtained from the National Oceanographic and Atmospheric Administrations’ (NOAA) Advanced Very High Resolution Radiometer [28]. These variables were included because many known contributors to cognitive dysfunction such as communicable and non-communicable diseases are influenced by environmental factors [22,25]. Other important environmental factors, such as land cover, land use, and soil were excluded due to unavailability of spatially referenced data. The environmental data used in this paper were extracted for the same year as the FLEMMS. Values of environmental variables at each survey location were extracted using geographic information system (GIS) software (ArcGIS version 10.4.0.5524) [29]. Electronic maps of the barangays were obtained from the geographic data warehouse DIVA GIS (www.diva-gis.org/Data) and PhilGIS (www.philgis.org).

### 2.3. Spatial Analysis

#### 2.3.1. Visualization of Spatial Heterogeneity of Prevalence of Functional Literacy Indicators

Functional illiteracy survey data were georeferenced to barangay centroids (longitude and latitude), which were estimated using the GIS software—Quantum GIS version 1.7.3 [30]. Spatial heterogeneity was assessed by using maps to visualise geographical variation of functional literacy indicators.

#### 2.3.2. Models of Prevalence of Functional Literacy Indicators

The prevalence of functional illiteracy levels in school-aged children aged between 10 and 19 years was modelled by transforming the original 2008 FLEMMS functional literacy levels into an ordinal variable with the following categories: functional illiteracy (cannot read, write, compute, or comprehend), low functional literacy (can only read and write), moderate functional literacy (can only read, write, and compute), and functional literacy (can read, write, compute, and comprehend). We used “functional literacy” as a reference group in our models. We developed a single frequentist fixed-effects multinomial regression model using the statistical software package, Stata version 13.1 [31] to assess associations between the levels of functional literacy and covariates for each region of the Philippines (i.e., Luzon, the Visayas, and Mindanao).

We built three nested models (Table A1). Model 1 included sociodemographic factors such as age, sex, level of education, socioeconomic status (SES) factors and water, sanitation, and hygiene (WASH) factors. Model 2 included the variables in Model 1 plus household education stimuli factors, and Model 3 included the variables in Model 2 plus environmental variables.

We built our models this way in order to disentangle the effects of individual, household, and environmental variables on the prevalence of functional literacy. Collinearity between covariates was measured using pairwise correlation coefficients, estimated in Stata version 13.1 [31]. Non-linearity in the univariate relationships between environmental variables and prevalence of functional illiteracy levels were assessed and adjusted for by adding quadratic terms where appropriate. Model fit was assessed using Akaike’s Information Criterion (AIC) [32]. Selection of candidate variables for the final multivariable model was conducted using univariable logistic regression models, with non-significant variables with Wald’s *p* > 0.2 excluded from further analysis. Backwards stepwise regression was conducted on the variables of the final multivariable model (Wald’s *p* < 0.05 as the entry criterion).

Marital status, employment, and occupation status were not found to be significantly associated with the prevalence of functional literacy in the preliminary multivariable models and thus were excluded from further analysis (Wald’s *p* > 0.2). We found collinearity between adult functional literacy rate and adult education attainment indicators, which provided support for the inclusion of adult functional literacy indicators only in our model.

We reported exponentiated coefficients in ratios of relative risks (RRR) which are equivalent to odds ratio in multinomial models. We transformed the estimated coefficients into RRR (e^b^) using a Stata command “*rrr*” in STATA [31].

#### 2.3.3. Analysis of Spatial Clustering in Functional Literacy Indicators

We used semivariograms to examine the presence of spatial autocorrelation in the observed prevalence of each level of functional literacy and in the residuals of the final multinomial models (residual semivariograms) at each barangay using the statistical package geoR of R software version 3.1.1 (The R foundation for statistical computing, Vienna, Austria, www.R-project.org). Semivariograms were generated for each functional literacy indicator for each region. A semivariogram is a graphical representation of the spatial variation in a dataset.

In the case of residual semivariograms, these represent the spatial variation left unexplained by the covariates included in a model. The semivariogram is characterised by three parameters: the partial sill, which is the spatially structured component of the semivariance (indicative of the tendency for geographical clustering), the nugget, which is the spatially unstructured component of the semivariance (representing random variation, very small-scale spatial variability, or measurement error) and the range, which is the distance at which locations can be considered independent (indicative of the average size of geographical clusters) [33]. Semivariograms of model residuals were compared between the three nested models.

## 3. Results

### 3.1. Dataset for Analysis

We analysed data from 11,313 school-aged children aged 10 to 19 years, including information on functional literacy, household, and demographic variables, and barangay geolocation. The average age of school-aged children was 13.6 years (Table 1).

The observed prevalence of functional illiteracy in school-aged children was most prevalent in the Visayas followed by Mindanao and Luzon (7.5%, 6.9%, and 3.0%, respectively; Table 1). The observed prevalence of functional illiteracy of heads of households was higher in the Visayas and Mindanao compared to Luzon (15.0%, 15.0%, and 6.1%, respectively) (Table 2). At the regional level, a higher proportion of households was classified as poor in Mindanao (42.6%) compared to the Visayas (38.6%) and Luzon (28.5%), *p* < 0.05) (Table 2). Luzon had higher average total education stimuli scores compared to Mindanao and the Visayas (*p* < 0.05) (Table 2 and Figure S2). Demographic characteristics of the heads of households are summarised in Table 2, and WASH characteristics are illustrated in Figure S3.

### 3.2. Spatial Distribution of Functional Literacy

**Figure 1:**
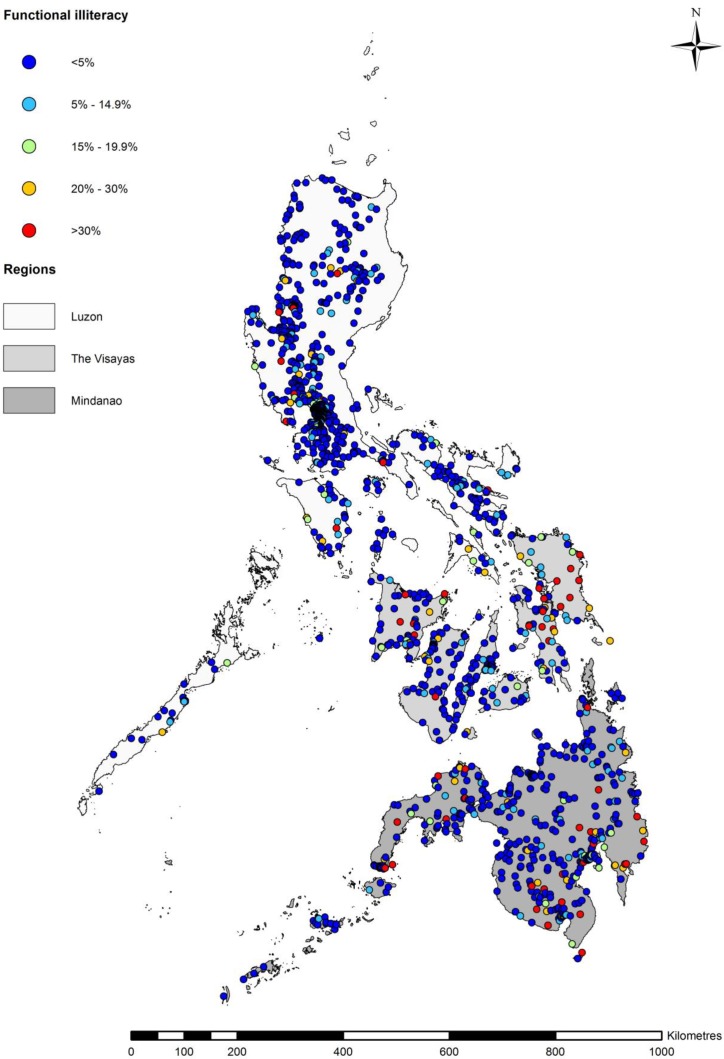
Map of prevalence of functional illiteracy in school-aged children.

A map of prevalence of functional illiteracy of school-aged children aged 10 to 19 years shows spatial heterogeneity between and within the three regions (Figure 1). Functional illiteracy of greater than 20% prevalence was observed across a wider geographic area in the Visayas and Mindanao compared to Luzon (Figure 1). The map suggests that in Luzon, a prevalence of functional illiteracy greater than 20% was observed in the northern part of the region, and prevalence greater than 15% was observed around the south-western part of Luzon (Figure 1). The map indicates that in the Visayas, a prevalence of functional illiteracy of greater than 30% was observed in the eastern part of the region. Within Mindanao, functional illiteracy was distributed towards the south, south-eastern, and south-western parts of the region (Figure 1).

Our maps illustrate that school-aged children with moderate and low functional literacy were spatially distributed across all regions of the Philippines (Figures S4 and S5).

**Figure 2:**
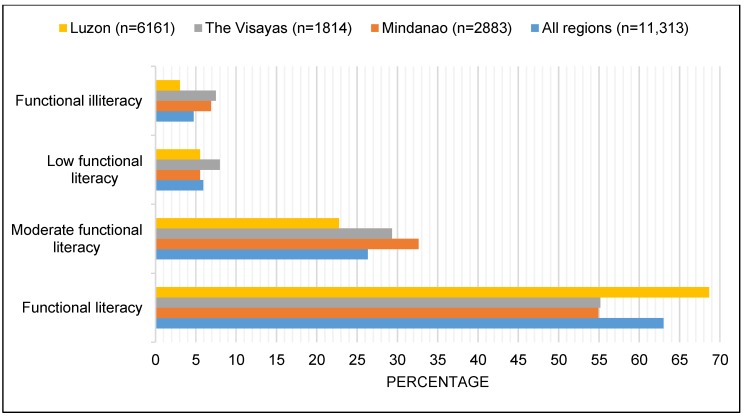
Prevalence of functional literacy in school-aged children in three regions of the Philippines.

Figure 2 shows that of the school-aged children who reside in Luzon (6616), 22.8% were classified as having moderate functional literacy, 5.5% were low functional literate, and 3% were functionally illiterate.

### 3.3. Models of Prevalence of Functional Literacy Indicators

For all regions, the model which included sociodemographic, SES, WASH, household education stimuli, and environmental variables (Model 3) fitted the data best (lowest AIC) [32], and the results for prevalence of functional illiteracy are summarised in Table 3. The results for prevalence of moderate functional literacy and prevalence of low functional literacy are summarised in Table 4 and Table 5.

Across all regions, age of school-aged children and illiteracy level of the heads of household were positively associated with the prevalence of all levels of functional literacy, except in Luzon where age was not associated with the prevalence of low functional literacy. Females (compared to males) were negatively associated with prevalence of functional illiteracy in Luzon and Mindanao (*p* < 0.001 and *p* = 0.03, respectively). Highest education attainment was negatively associated with prevalence of all levels of functional literacy in all regions (between *p* = 0.05 and *p* < 0.001), except in the Visayas where this association was observed only for the prevalence of functional illiteracy (*p* < 0.001).

High SES was negatively associated with low functional literacy (*p* < 0.001) (Table 5). ‘Lake or pond or rainwater and rivers’ as main source of drinking water compared to ‘piped into dwelling’ (*p* < 0.001), and household education stimuli score were negatively associated with functional illiteracy (ratio of relative risks [RRR], 0.92; *p* = 0.01). Households in Luzon without access to toilets were positively associated with functional illiteracy compared to households with flush toilets ([RRR], 2.26; *p* = 0.03) (Table 3).

In the Visayas, 29.3% of school-aged children were classified as having moderate functional literacy, 8% were low functional literate, and 7.5% were functionally illiterate (Figure 2). Protected well as a source of drinking water (*p* = 0.01), and LST were negatively associated with moderate functional literacy (*p* = 0.02); household education stimuli was negatively associated with moderate and low functional literacy (both *p* = 0.03). SES was negatively associated with functional illiteracy, albeit non-significant (*p* = 0.07).

In Mindanao, 32.6% of school-aged children were classified as having moderate functional literacy, 5.5% were low functional literate, and 6.9% were functionally illiterate (Figure 2). Household education stimuli was negatively associated with low functional literacy (*p* = 0.05), and DPWB was positively associated with low functional literacy (*p* < 0.001). SES was negatively associated with functional illiteracy ([RRR], 0.50; *p* = 0.01). DPWB ([RRR], 1.25; *p* = 0.02) and NDVI ([RRR], 1.42; *p* = 0.01) was positively associated with functional illiteracy, while rainfall ([RRR], 0.73; *p* = 0.01) and LST ([RRR], 0.75; *p* = 0.05) were negatively associated.

### 3.4. Spatial Dependence in Functional Literacy Indicators

**Figure 3:**
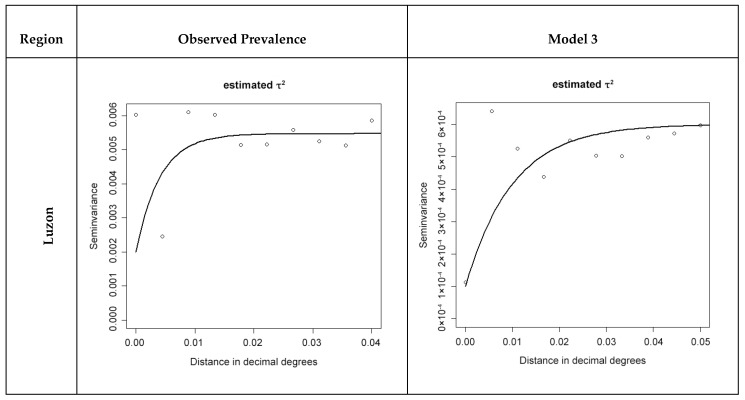

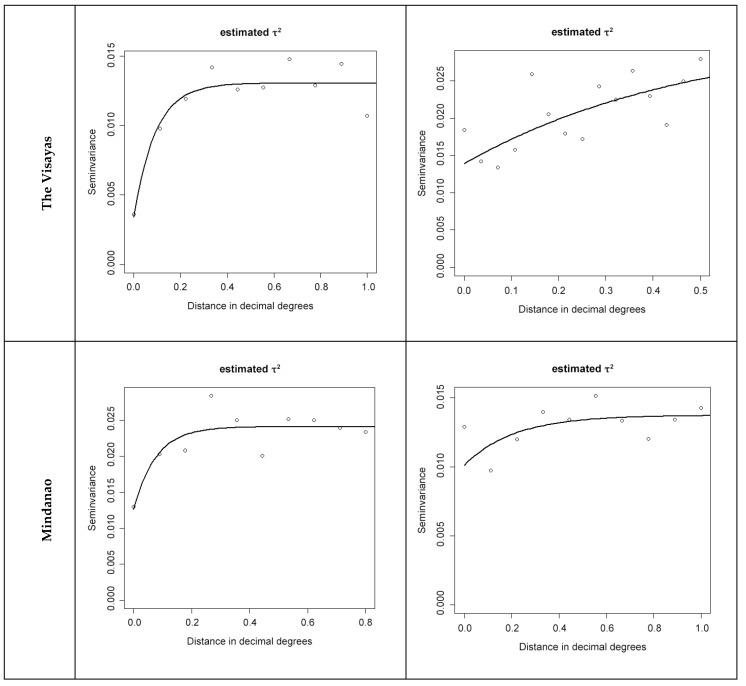
Semivariograms of prevalence of functional illiteracy in school-aged children.

Our results indicate spatial dependency in the prevalence of all three functional literacy indicators (Figure 3 and Figures S6–S8).

Across all regions, we observed larger cluster size after adjusting for the effect of covariates. In Luzon, 52% of the variance in moderate functional literacy was spatially structured with an average cluster size of 1.2 km. After adjusting for the effect of covariates in Model 3, the percentage of overall variance that was spatially structured and the average size of clusters of moderate functional literacy were 64% and 1.7 km respectively (Table S2). For low functional literacy, 95.3% of the variance was spatially structured with an average cluster size of 0.7 km. After adjusting for the effect of covariates in Model 3, the percentage of overall variance that was spatially structured and the average size of clusters of low functional literacy were 83% and 3.3 km respectively (Table S3). For functional illiteracy, 63.3% of the variance in functional illiteracy was spatially structured with an average cluster size of 1.3 km. After adjusting for the effect of covariates in Model 3, 83% of the variation was spatially structured and the average size of cluster was 3.3 km (Table 6).

In the Visayas, 73.8% of the variance in functional illiteracy was spatially structured with an average cluster size of 30.7 km (Table 6). After adjusting for the covariates, residual spatial dependency of functional illiteracy was no longer visible in that the semivariograms of all three models showed a spatial trend in the prevalence of functional illiteracy but no clustering. Our findings demonstrate spatial dependency only in the raw prevalence of functional illiteracy (Figure 3).

In Mindanao, 27.4% of the variance in moderate functional literacy was spatially structured with an average cluster size of 31.3 km. After adjusting for the effect of covariates in Model 3, 3.4% of the variation was spatially structured, the average size of cluster was unchanged (Table 6), and 48% of the variance in functional illiteracy was spatially structured with an average cluster of 25 km. After adjusting for the effect of covariates included in Model 3, 26.2% of the variance was spatially structured, and the average size of cluster was 69.47 km (Table 6). Our results indicated spatial dependency in the prevalence of moderate functional literacy (Figure S7), and functional illiteracy (Figure 3).

## 4. Discussion

Functional literacy is a key indicator of cognitive function, especially information processing and comprehension, and has been used to measure cognitive function in school-aged children in previous studies [15,34,35,36,37]. Evaluation of geographical determinants of functional literacy is critical for designing and implementing spatially targeted interventions which could contribute to efficiently attaining the Sustainable Development Goals (SDGs) for functional literacy in school-aged children in the Philippines [38].

This study represents the first nation-wide population based spatial epidemiological study investigating geographical disparities in functional literacy indicators and its association with individual-level variables (sociodemographic factors), household-level variables (SES, WASH, household education stimuli factors), and environmental variables (DPWB, LST, NDVI, rainfall). Our study indicates that the prevalence of functional illiteracy is heterogeneous in the Philippines while showing important regional differences in key determinants of functional illiteracy.

### 4.1. The Role of Household WASH and Educational Stimuli in the Spatial Variation of Functional Illiteracy in Luzon

Household WASH variables including type of toilet facility and the main sources of drinking water were found to be important determinants of the prevalence of functional illiteracy in the region of Luzon. Households in Luzon without access to toilets had higher prevalence of functional illiteracy compared to households with flush toilets.

Previous studies have demonstrated that practice of open defecation increases the risk of childhood stunting, and transmission of infectious diseases [39,40]. Available evidence suggests that households with access to unprotected drinking water sources are at increased risk of infectious diseases such as cholera, and other bacterial and infectious diarrhoeal diseases which are also known to affect children’s development [41,42]. Our results show that the use of unprotected drinking water sources (lake, pond, rainwater or rivers) is also associated with reduced functional illiteracy.

Our findings may be confounded by the fact that unprotected drinking water sources are more likely to be present in agricultural communities where access to food and nutritional security are maintained through local food production. Natural rivers and lakes are often used as sources of agricultural irrigation and water for livestock [43]. Indeed, previous studies indicate that food security has a positive impact on nutritional status and the long-term cognitive development of children [44]. Further investigation is needed to examine the factors mediating the relation between access to water sources and the prevalence of functional illiteracy identified in this study.

Our results also demonstrate that in Luzon, the quality of the home environment is an important predictor of functional literacy of school-age children. Indeed, our results showed that of the three regions, Luzon had the lowest proportion of households classified as poor and that the average total scores of households’ education stimuli were higher in Luzon compared to the Visayas and Mindanao. This may mean that household education stimuli available to children in Luzon reflects not only household SES but that there is variability of household education stimuli scores within SES classes. Previous studies also found house environment mediated the association between family SES and executive functions of inhibitory control and working memory in school-aged children [45].

Taken together, our findings suggest that functional literacy in Luzon may benefit from health promotion interventions that improve personal hygiene practices, which could play a key role in mediating the effects of infectious diseases and malnutrition, and assist families with providing a stimulating environment for children of school age.

### 4.2. The Role of Environment and Socioeconomic Status (SES) in the Spatial Variation of Functional Illiteracy in the Visayas and Mindanao

Environmental factors and poverty play important roles in the spatial variation of functional illiteracy in the Visayas and Mindanao. While our results indicate that environmental determinants play different roles in functional illiteracy depending on the region, the effects of environmental variables on the spatial variation of functional illiteracy were greatest in Mindanao.

The Philippines frequently experiences droughts, typhoons, and flooding which lead to the destruction of crops, land degradation, and siltation of irrigation systems caused by severe erosion [46]. Such extreme weather events are of concern particularly among households in Mindanao where agriculture and fisheries are the major economic activities. Evidence suggests that since early 2000 extreme weather events including long periods of high temperatures and intense rainfall have intensified in Mindanao [47]. Farming communities are particularly vulnerable to climate-associated food insecurity which has a negative impact on the nutritional status and child development in affected populations, as well as increasing the susceptibility to disease [44,47].

Our findings showed that low SES was positively associated with functional illiteracy in Mindanao. Evidence suggests a possible link (behavioural and neurobiological) between low SES and functioning of different domains of neurocognitive systems including children’s performance on language and literacy skills [19,48]. This relation is mediated by different mechanism such as parenting behaviour, linguistic stimulation and children’s experience of stress [19].

The observed association could also be explained by the effect of malnutrition in the poorest areas of Mindanao. Our map of the spatial distribution of functional illiteracy in Mindanao (Figure 1) shows a higher rate of functional illiteracy in western Mindanao, known for its high prevalence of underweight, stunting, and wasting in children [49,50]. Furthermore, Mindanao has suffered social and political conflict for many years which have led to greater food insecurity, limited land use options, and a higher rate of poverty in this region [47]. Further investigation is needed to understand the factors that mediate the association between SES and functional illiteracy in Mindanao and the Visayas.

Taken together, our findings suggest that poverty is likely to be a key determinant of malnutrition in this region, explaining the geographical heterogeneity of functional illiteracy indicators. Areas with high rates of poverty may benefit from integrated interventions that aim to reduce malnutrition in school-aged children.

### 4.3. Limitations

The results of this study need to be interpreted in light of several limitations. Our estimates of functional illiteracy indicators rely on performance-based functional literacy data. Although these tools are designed to measure different domains of cognitive functioning in school-age children, performance-based measurement tools may be differentially related to the outcome—for example participants who completed self-reported questionnaires may have had a chance to ask interviewers questions and get support when required [51].

Further, our data are from the 2008 FLEMMS survey and may not reflect the current situation. However, these data constitute the most comprehensive and up to date information on functional illiteracy in the Philippines and yield novel insights into the ways environmental input can affect child development. Data on literacy rates are scarce due to the costs involved in their collection and processing [8] and in the Philippines, census data are collected every ten years [7]. The latest data on literacy were collected in 2010, however information on functional literacy rate has not been updated in the report produced by the Philippines government since 2008 [6,7]. It is notable, however, that the rate of functional literacy has not seen much improvement in the last three FLEMMS surveys (83.8% in 1994, 84.1% in 2003, and 86.4% in 2008 (data from 1989 was not available for comparison)) [7] and therefore we do not expect much difference in the current situation.

In addition, we used an ecological approach, using secondary data on environmental predictors of functional illiteracy such as climate and SES [44,52]. Some of these proxies are imprecise measurements of exposure, resulting in regression dilution bias leading to underestimation of the observed effects [53].

Further, we created our own home inventory-proxy measurement (education stimuli measure), so our results cannot be readily compared with the standard home-inventory index questionnaire [54,55,56]. Our education stimuli measure included selected variables in the FLEMMS survey, which attempt to document whether access to learning materials and activities would improve cognitive function of school-aged children [57,58,59]. Nevertheless, our results indicated that there is evidence for a degree of specificity of the effect of household education stimuli. Our education stimuli score appeared to be a reasonably reliable scale with moderate correlations with SES element, which was based on ownership of household amenities (a pairwise correlation coefficient of 0.36).

Finally, our paper highlights the need of a complementary modelling approach that could be used to investigate potential reverse causation. While standard multivariate regression models may be useful for exploring the effects of predictor variables on the outcome variable, it may not be sufficient to explain the interrelation (associations and dependency) between multiple interdependent variables [60,61]. Previous studies showed that maternal literacy can improve the health navigation skills of mothers, which leads to child mortality reduction [34,62]. Multiple determinants of literacy have variable impact depending on where and how they are embedded in a child’s life. Future studies need to account for the complex interdependencies between these determinants.

## 5. Conclusions

Our study demonstrates that functional literacy is heterogeneous in the Philippines and that the determinants of functional literacy vary between regions. The results support the need for geographically targeted interventions that consider the context-specific determinants identified in this study. In the context of the current work, this is particularly relevant in order for the Philippines to achieve the Sustainable Development Goals for literacy by 2030. More broadly, our findings demonstrate an approach that can guide policy makers elsewhere, to design geographically targeted intervention programs for populations most in need.

## Figures and Tables

**Table 1:** Demographic characteristics of school-aged children, stratified by regions of the Philippines.

Variables		Regions		
		Luzon (*n* = 6616)	The Visayas (*n* = 1814)	Mindanao (*n* = 2883)
Age	Mean age (Standard deviation)	13.5 (2.37)	13.6 (2.46)	13.8 (2.50)
Sex	Male	3427 (51.8)	924 (50.9)	1485 (51.5)
	Female	3189 (48.2)	890 (49.1)	1398 (48.5)
Functional literacy level	Functional literacy	4544 (68.7)	1001 (55.2)	1584 (54.9)
	Moderate functional literacy	1506 (22.8)	532 (29.3)	941 (32.6)
	Low functional literacy	366 (5.5)	145(8.0)	159 (5.5)
	Functional illiteracy	200 (3.0)	136 (7.5)	199 (6.9)
Highest education attainment	No grade completed	33 (0.5)	24 (1.3)	58 (2.0)
	Elementary level	3765 (56.9)	1079 (59.5)	1730 (60)
	High school level	2818 (42.6)	711 (39.2)	1095 (38)
Employment status	Yes	689 (10.0)	314 (17.3)	472 (16.4)
	No	5927 (90.0)	1500 (82.7)	2411 (83.6)

Note: Unless otherwise indicated, values represent the absolute number followed by the percentage within parentheses.

**Table 2:** Demographic characteristics of the heads of households and household socioeconomic status and education stimuli score, stratified by regions of the Philippines.

Variables		Regions		
		Luzon (*n* = 5791)	The Visayas (*n* = 1673)	Mindanao (*n* = 2875)
Age	Mean age (Standard deviation)	13.5 (2.37)	13.6 (2.46)	13.8 (2.50)
Sex	Male	3427 (51.8)	924 (50.9)	1485 (51.5)
	Female	3189 (48.2)	890 (49.1)	1398 (48.5)
Marital status	Single	842 (14.5)	286 (17.1)	487 (16.9)
	Married	4454 (76.9)	1286 (75.8)	2210 (76.9)
	Widow	349 (6)	95 (5.7)	130 (4.5)
	Divorced	146 (2.5)	24 (1.4)	48 (1.7)
Functional literacy level	Functional literacy	4544 (68.7)	1001 (55.2)	1584 (54.9)
	Moderate functional literacy	1506 (22.8)	532 (29.3)	941 (32.6)
	Low functional literacy	366 (5.5)	145(8)	159 (5.5)
	Functional illiteracy	200 (3)	136 (7.5)	199 (6.9)
Highest education attainment	No grade completed	33 (0.5)	24 (1.3)	58 (2)
	Elementary level	3765 (56.9)	1079 (59.5)	1730 (60)
	High school level	2818 (42.6)	711 (39.2)	1095 (38)
Occupation	Worked for private household	1787 (30.8)	485 (29)	707 (24.6)
	Worked for government	94 (1.6)	33 (2)	72 (2.5)
	Worked with pay on own family-operated Farm or business	1890 (32.6)	647 (38.7)	1037 (36.1)
	Worked without pay on own family-operated farm or business	544 (9.4)	189 (11.3)	395 (13.7)
	Unemployed	1476 (25.6)	319 (19)	664 (23.1)
Socioeconomic status (SES)	Poor—Low SES	1925 (33.2)	713 (42.6)	1434 (49.9)
	Non-poor—High SES	3866 (66.8)	960 (57.4)	1441 (50.1)
Household education stimuli score	Average Score (Standard deviation)	8.12 (3.28)	7.56 (3.14)	7.03 (3.33)
	95% Confidence Interval	8.04, 8.20	7.01, 7.71	6.91, 7.15

Note: Unless otherwise indicated, values represent the absolute number followed by the percentage within parentheses.

**Table 3:** Association of selected covariates with functional illiteracy in school-aged children in the Philippines.

Covariates		Luzon		The Visayas		Mindanao	
		RRR ^a^ (95CI ^b^)	*p*-Value	RRR ^a^ (95CI ^b^)	*p*-Value	RRR ^a^ (95CI ^b^)	*p*-Value
Sociodemographic	Age in years (continuous)	1.01 (0.92, 1.10)	0.90	1.90 (0.99, 1.22)	0.06	1.09 (0.98, 1.19)	0.09
	Female (vs. Male)	0.47 (0.33, 0.66)	***	0.71 (0.47, 1.08)	0.11	0.61 (0.39, 0.950)	*
	Elementary level (vs. No grade completed)	0.01 (0.00, 0.05)	***	0.05 (0.01, 0.16)	***	1.36 × 10^−8^ (3.07 × 10^−9^, 6.01 × 10^−8^)	***
	High school level (vs. No grade completed)	0.002 (0.00, 0.01)	***	0.01 (0.00, 0.01)	***	1.63 × 10^−9^ (3.42 × 10^−10^, 7.83 × 10^−9^)	***
	Mean functional illiteracy levels of the heads of household	2.58 (1.86, 3.58)	***	4.74 (3.05, 7.38)	***	6.29 (3.94, 10.08)	***
SES ^c^	High SES (vs. Low SES)	1.09 (0.72, 1.63)	0.69	0.66 (0.42, 1.04)	0.07	0.50 (0.31, 0.82)	**
Main sources of drinking water for members of household	Protected well (vs. Piped into dwelling)	0.89 (0.63, 1.27)	0.53	1.43 (0.90, 2.57)	0.22	1.01 (0.59, 1.70)	0.97
	Unprotected well (vs. Piped into dwelling)	1.15 (0.55, 2.45)	0.72	1.45 (0.49, 4.24)	049	0.39 (0.15, 1.07)	0.07
	Lake/pond/rain water/rivers (vs. Piped into dwelling)	1.09 × 10^−9^ (3.30 × 10^−10^, 3.60 × 10^−9^)	***	1.36 (0.50, 3.69)	0.99	2.06 (0.47, 8.99)	0.34
	Other (vs. Piped into dwelling)	2.89 (0.87, 9.67)	0.08	1.36 (0.50, 3.69)	0.54	0.51 (0.19, 1.34)	0.17
The types of toilet facility at home	Pit toilet (vs. Flush toilet)	0.98 (0.50, 1.92)	0.95	0.42 (0.14, 1.27)	0.12	0.66 (0.29, 1.50)	0.32
	No toilet/bush/field (vs. Flush toilet)	2.26 (1.19, 4.27)	**	1.49 (0.65, 3.45)	0.34	0.44 (0.15, 1.29)	0.14
Main material of floor	Bamboo/palm/wood floor (vs. Natural floor earth/sand)	0.65 (0.33, 1.29)	0.22	1.45 (0.66, 3.45)	0.35	0.96 (0.48, 1.95)	0.92
	Cement floor (vs. Natural floor earth/sand)	0.74 (0.44, 1.25)	0.26	0.76 (0.35, 1.64)	0.48	0.50 (0.21, 1.17)	0.11
	Other floor (vs. Natural floor earth/sand)	0.78 (0.39, 1.53)	0.46	1.55 (0.66, 3.66)	0.32	0.79 (0.27, 2.31)	0.66
Main material of outer walls of houses	Aluminium walls (vs. Bamboo/palm/wood)	0.89 (0.61, 1.32)	0.59	1.39 (0.72, 2.67)	0.32	1.39 (0.66, 2.92)	0.39
	Other walls (vs. Bamboo/palm/wood)	0.52 (0.20, 1.31)	0.16	1.21 (0.38, 3.89)	0.75	1.07 (0.34, 3.39)	0.91
	Household education stimuli mean score	0.92 (0.87, 0.98)	**	1.02 (0.93, 1.11)	0.75	0.96 (0.89, 1.04)	0.29
Environment	DPWB ^d^	1.12 (0.87, 1.42)	0.38	0.80 (0.59, 1.08)	0.71	1.25 (1.02, 1.53)	*
	DPWB^2	0.84 (0.74, 0.95)	0.01	1.03 (0.88, 1.19)	0.72	N/A	N/A
	LST ^e^	0.97 (0.77, 1.21)	0.78	0.84 (0.49, 1.43)	0.52	0.75 (0.56, 0.99)	*
	LST^2	0.99 (0.97, 1.02)	0.62	0.89 (0.77, 1.04)	0.13	N/A	N/A
	NDVI ^f^	0.94 (0.65, 1.34)	0.71	1.74 (0.98, 3.09)	0.06	1.42 (1.09, 1.84)	**
	NDVI^2	1.02 (0.93, 1.11)	0.68	1.59 (1.12, 2.25)	0.01	N/A	N/A
	Rain	1.27 (0.87, 1.85)	0.22	1.10 (0.72, 1.69)	0.65	0.73 (0.58, 0.92)	**
	Rain^2	0.96 (0.87, 1.06)	0.40	0.84 (0.67, 1.05)	0.12	N/A	N/A
	Intercept	10.12 (1.29, 79.37)	0.03	0.16 (0.02, 1.54)	0.11	13.75 (11.87, 15.62)	0.00

Note: Reference group = functional literacy. ^a^ RRR = ratios of relative risks; ^b^ 95CI = 95% confidence interval; ^c^ SES = socioeconomic status; ^d^ DPWB = distance to perennial water body; ^e^ LST = land surface temperature; ^f^ NDVI = normalised difference vegetation index; * = statistically significant (*p* < 0.05); ** = statistically significant (*p* < 0.01); *** = statistically significant (*p* < 0.001).

**Table 4:** Association of selected covariates with moderate functional literacy in school-aged children in the Philippines (Model 3).

Covariates		Luzon		The Visayas		Mindanao	
		RRR ^a^ (95CI ^b^)	*p*-Value	RRR ^a^ (95CI ^b^)	*p*-Value	RRR ^a^ (95CI ^b^)	*p*-Value
Sociodemographic	Age in years (continuous)	1.09 (1.05, 1.12)	***	1.05 (0.99, 1.12)	0.07	1.09 (1.05, 1.14)	***
	Female (vs. Male)	0.79 (0.79, 0.89)	***	0.99 (0.78, 1.28)	0.98	0.76 (0.64, 0.89)	***
	Elementary level (vs. No grade completed)	0.26 (0.07, 1.02)	*	0.33 (0.10, 1.09)	0.07	2.21 × 10^−6^ (2.63 × 10^−7^,0.00)	***
	High school level (vs. No grade completed)	0.15 (0.04, 0.57)	**	0.21 (0.06, 0.71)	**	9.57 × 10^−7^ (1.21 × 10^−7^,7.55 × 10^−6^)	***
	Mean functional illiteracy levels of the heads of household	1.81 (1.55, 2.12)	***	1.34 (1.04, 1.73)	*	2.24 (1.73, 2.89)	***
SES ^c^	High SES (vs. Low SES)	0.92 (0.77, 1.10)	0.38	0.84 (0.64, 1.12)	0.24	0.96 (0.75, 1.23)	0.73
Main sources of drinking water for members of household	Protected well (vs. Piped into dwelling)	1.04 (0.89, 1.21)	0.61	0.65 (0.48, 0.89)	**	1.16 (0.87, 1.54)	0.30
	Unprotected well (vs. Piped into dwelling)	1.02 (0.72, 1.46)	0.89	0.73 (0.45, 1.19)	0.21	0.96 (0.75, 1.23)	0.84
	Lake/pond/rain water/rivers (vs. Piped into dwelling)	0.83 (0.42, 1.63)	0.59	1.33 (0.64, 1.12)	0.45	0.83 (0.28, 2.44)	0.74
	Other (vs. Piped into dwelling)	1.45 (0.92, 2.29)	0.11	1.06 (0.50, 2.23)	0.88	0.69 (0.39, 1.20)	0.19
The types of toilet facility at home	Pit toilet (vs. Flush toilet)	0.84 (0.61, 1.14)	0.26	0.54 (0.26, 1.16)	0.11	1.01 (0.67, 1.54)	0.95
	No toilet/bush/field (vs. Flush toilet)	1.09 (0.77, 1.55)	0.61	0.84 (0.55, 1.29)	0.43	1.07 (0.67, 1.72)	0.78
Main material of floor	Bamboo/palm/wood floor (vs. Natural floor earth/sand)	1.02 (0.75, 1.39)	0.92	1.02 (0.67, 1.54)	0.94	1.29 (0.85, 1.99)	0.23
	Cement floor (vs. Natural floor earth/sand)	1.09 (0.85, 1.41)	0.48	1.07 (0.64, 1.79)	0.79	1.09 (0.69, 1.72)	0.71
	Other floor (vs. Natural floor earth/sand)	1.04 (0.76, 1.41)	0.81	1.08 (0.56, 2.09)	0.82	0.98 (0.54, 1.78)	0.96
Main material of outer walls of houses	Aluminium walls (vs. Bamboo/palm/wood)	0.85 (0.70, 1.02)	0.09	1.02 (0.67, 1.54)	0.41	1.06 (0.77, 1.46)	0.73
	Other walls (vs. Bamboo/palm/wood)	0.81 (0.58, 1.14)	0.23	0.90 (0.49, 1.64)	0.74	1.01 (0.57, 1.77)	0.98
	Household education stimuli mean score	0.95 (0.93, 0.98)	***	0.95 (0.91, 0.99)	*	0.98 (0.95, 1.03)	0.51
Environment	DPWB ^d^	1.05 (0.94, 1.16)	0.43	1.12 (0.92, 1.38)	0.26	1.10 (0.96, 1.31)	0.16
	DPWB^2	1.02 (0.98, 1.07)	0.36	1.01 (0.93, 1.10)	0.75	N/A	N/A
	LST ^e^	1.02 (0.91, 1.14)	0.79	0.70 (0.53, 0.93)	*	0.89 (0.76, 1.05)	0.16
	LST^2	0.99 (0.98, 1.01)	0.55	0.94 (0.89, 0.98)	**	N/A	N/A
	NDVI ^f^	0.95 (0.93, 0.98)	0.59	0.98 (0.69, 1.41)	0.93	1.13 (0.97, 1.31)	0.12
	NDVI^2	0.98 (0.92, 1.03)	0.41	1.20 (0.94, 1.54)	0.14	N/A	N/A
	Rain	0.93 (0.79, 1.09)	0.35	1.03 (0.78, 1.36)	0.82	1.00 (0.85, 1.19)	0.95
	Rain^2	1.04 (0.99, 1.09)	0.08	1.00 (0.89, 1.12)	0.97	N/A	N/A
	Intercept	0.68 (0.16, 2.92)	0.59	0.95 (0.20, 4.49)	0.95	10.07 (8.52, 12.93)	0.00

Note: Reference group = functional literacy. ^a^ RRR = ratios of relative risks; ^b^ 95CI = 95% confidence interval; ^c^ SES = socioeconomic status; ^d^ DPWB = distance to perennial water body; ^e^ LST = land surface temperature; ^f^ NDVI = normalised difference vegetation index; * = statistically significant (*p* < 0.05); ** = statistically significant (*p* < 0.01); *** = statistically significant (*p* < 0.001).

**Table 5:** Association of selected covariates with low functional literacy in school-aged children in the Philippines (Model 3).

Covariates		Luzon		The Visayas		Mindanao	
		RRR ^a^ (95CI ^b^)	*p*-Value	RRR ^a^ (95CI ^b^)	*p*-Value	RRR ^a^ (95CI ^b^)	*p*-Value
Sociodemographic	Age in years (continuous)	1.06 (0.99, 1.13)	0.08	1.01 (0.90, 1.34)	0.82	0.96 (0.88, 1.04)	0.32
	Female (vs. Male)	0.70 (0.56, 0.88)	***	0.65 (0.42, 1.01)	0.06	0.79 (0.57, 1.08)	0.14
	Elementary level (vs. No grade completed)	0.14 (0.26, 0.73)	*	0.86 (0.09. 8.21)	0.89	1.80 × 10^−7^ (3.30 × 10^−8^,9.79 × 10^−7^)	***
	High school level (vs. No grade completed)	0.06 (0.01, 0.33)	***	0.29 (0.03, 2.87)	0.29	5.80 × 10^−8^ (9.97 × 10^−9^,3.37 × 10^−7^)	***
	Mean functional illiteracy levels of the heads of household	3.07 (2.28, 4.12)	***	4.43 (2.98, 6.57)	***	3.12 (2.04, 4.78)	***
SES ^c^	High SES (vs. Low SES)	0.66 (0.49, 1.62)	***	0.69 (0.44, 1.09)	0.11	0.82 (0.53, 1.27)	0.36
Main sources of drinking water for members of household	Protected well (vs. Piped into dwelling)	1.25 (0.94, 1.66)	0.13	1.13 (0.68, 1.86)	0.64	1.12 (0.71, 1.78)	0.63
	Unprotected well (vs. Piped into dwelling)	0.88 (0.49, 1.62)	0.69	0.41 (0.68, 1.86)	0.12	0.91 (0.46, 1.79)	0.79
	Lake/pond/rain water/rivers (vs. Piped into dwelling)	2.63 (0.38, 18.16)	0.33	0.56 (0.17, 1.86)	0.34	0.91 (0.46, 1.79)	0.07
	Other (vs. Piped into dwelling)	0.99 (0.26, 3.79)	0.99	0.88 (0.25, 3.02)	0.83	3.04 (0.46, 1.79)	0.11
The types of toilet facility at home	Pit toilet (vs. Flush toilet)	1.43 (0.87, 2.36)	0.16	0.31 (0.08, 1.13)	0.08	1.78 (0.94, 3.39)	0.08
	No toilet/bush/field (vs. Flush toilet)	1.64 (0.88, 3.05)	0.12	0.60 (0.28, 1.25)	0.17	1.25 (0.55, 2.84)	0.59
Main material of floor	Bamboo/palm/wood floor (vs. Natural floor earth/sand)	0.75 (0.43, 1.32)	0.31	1.09 (0.56, 2.15)	0.78	1.00 (0.45, 2.22)	0.99
	Cement floor (vs. Natural floor earth/sand)	1.02 (0.66, 1.59)	0.92	0.86 (0.38, 1.96)	0.73	0.86 (0.37, 1.99)	0.72
	Other floor (vs. Natural floor earth/sand)	1.06 (0.63, 1.79)	0.83	1.23 (0.77, 2.04)	0.69	1.30 (0.48, 3.49)	0.60
Main material of outer walls of houses	Aluminium walls (vs. Bamboo/palm/wood)	0.93 (0.66, 1.32)	0.69	1.25 (0.77, 2.04)	0.36	1.27 (0.73, 2.19)	0.39
	Other walls (vs. Bamboo/palm/wood)	1.06 (0.59, 1.89)	0.83	0.41 (0.12, 1.38)	0.15	2.02 (0.83, 4.89)	0.12
	Household education stimuli mean score	0.89 (0.85, 0.94)	***	0.85 (0.79, 0.93)	***	0.93 (0.87, 1.00)	*
Environment	DPWB ^d^	1.05 (0.85, 1.30)	0.66	1.42 (0.86, 2.33)	0.17	1.49 (1.18, 1.87)	***
	DPWB^2	0.93 (0.81, 1.07)	0.32	0.87 (0.76, 1.01)	0.07	NA	NA
	LST ^e^	1.06 (0.89, 1.27)	0.50	0.71 (0.38, 1.37)	0.32	0.83 (0.67, 1.04)	0.11
	LST^2	0.99 (0.98, 1.01)	0.65	0.94 (0.84, 1.04)	0.24	NA	NA
	NDVI ^f^	0.93 (0.67, 1.28)	0.64	0.96 (0.57, 1.61)	0.87	1.07 (0.85, 1.36)	0.56
	NDVI^2	1.04 (0.94, 1.14)	0.45	1.13 (0.67, 1.92)	0.64	NA	NA
	Rain	1.02 (0.77, 1.35)	0.88	1.13 (0.53, 2.41)	0.74	1.03 (0.82, 1.28)	0.82
	Rain^2	0.98 (0.91, 1.05)	0.56	0.77 (0.55, 1.07)	0.12	NA	NA
	Intercept	0.66 (0.08, 5.11)	0.69	0.24 (0.01, 4.26)	0.33	13.09 (11.24, 14.94)	0.00

Note: Reference group = functional literacy. ^a^ RRR = ratios of relative risks; ^b^ 95CI = 95% confidence interval; ^c^ SES = socioeconomic status; ^d^ DPWB = distance to perennial water body; ^e^ LST = land surface temperature; ^f^ NDVI = normalised difference vegetation index; * = statistically significant (*p* < 0.05); ** = statistically significant (*p* < 0.01); *** = statistically significant (*p* < 0.001).

**Table 6:** Results of semivariograms for prevalence of functional illiteracy and moderate and low functional literacy.

Indicators	Functional Illiteracy		Moderate Functional Literacy		Low Functional Literacy	
	Observed Data	Model 3	Observed Data	Model 3	Observed Data	Model 3
**Luzon**						
Partial sill	0.0035	0.0005	0.0295	0.0069	0.0271	0.002
Nugget	0.002	0.0001	0.0271	0.0039	0.0013	0.0004
Practical range (km) ^a^	0.012 (1.33)	0.03 (3.33)	0.011 (1.22)	0.015 (1.67)	0.006 (0.67)	0.03 (3.33)
% of the variance due to clustering ^b^	63.62	83.33	52.16	63.72	95.28	83.02
**The Visayas**						
Partial sill	0.0096	0.0176	0.05	0.0019	0.005	0.0011
Nugget	0.0034	0.0139	0.04	0.0051	0.001	0.0017
Practical range (km) ^a^	0.277 (30.74)	1.444 (160.28)	0.0001 (0.01)	0.093 (10.32)	0.000 (0.01)	0.626 (69.49)
% of the variance due to clustering ^b^	73.83	55.86	55.56	27.50	83.33	39.81
**Mindanao**						
Partial sill	0.0115	0.0036	0.0224	0.0005	0.0006	0.0003
Nugget	0.0126	0.0101	0.0593	0.0128	0.0099	0.0003
Practical range (km) ^a^	0.229 (25.42)	0.632 (69.47)	0.282 (31.30)	0.279 (30.97)	1.645 (180.98)	0.299 (33.19)
% of the variance due to clustering ^b^	47.66	26.23	27.37	3.44	5.93	50.00

Note: ^a^ Calculation based on practical range multiplied by 111. 1 decimal degree = 111 km, 0.1 = 11 km, 0.01 = 1 km, 0.05 = 5 km, 0.005 = 500 m; ^b^ Calculation based on partial sill divided by sill (partial sill and nugget), multiplied by 100.

## Supplementary Materials

The following are available online at http://www.mdpi.com/1660-4601/16/1/137/s1: Text S1: Sampling methods of the Functional Literacy, Education, and Mass Media Survey (FLEMMS); Text S2: Results of semivariograms for prevalence of moderate functional literacy; Table S1: Household education stimuli score, Table S2: Results of semivariograms for prevalence of moderate functional literacy, Table S3: Results of semivariograms for prevalence of low functional literacy; Figure S1: Map of 2008 FLEMMS survey locations; Figure S2: Box plot showing the relationship between household education stimuli average total score and functional literacy indicators, by region; Figure S3: Bar graph showing basic household WASH characteristics; Figure S4: Maps of prevalence of moderate functional literacy of school-aged children in the Philippines by region; Figure S5: Maps of prevalence of low functional literacy of school-aged children in the Philippines by region; Figure S6: Semivariograms of prevalence of functional illiteracy in school-aged children; Figure S7: Semivariograms of prevalence of moderate functional literacy in school-aged children; Figure S8: Semivariograms of prevalence of low functional literacy in school-aged children.

## Appendix A

**Table A1:** List of covariates included in the models.

Models	Covariates Included in the Model				
	Sociodemographic	SES	WASH	Household Education Stimuli	Environment
Model 1	✓	✓	✓	✗	✗
Model 2	✓	✓	✓	✓	✗
Model 3	✓	✓	✓	✓	✓

## References

[1] UNESCO Institute for Statistics 50th Anniversary of international literacy day: Literacy rates are on the rise but millions remain illiterate. http://uis.unesco.org/sites/default/files/documents/fs38-50th-anniversary-of-international-literacy-day-literacy-rates-are-on-the-rise-but-millions-remain-illiterate-2016-en.pdf.

[2] Noble K.G., Houston S.M., Brito N.H., Bartsch H., Kan E., Kuperman J.M., Akshoomoff N., Amaral D.G., Bloss C.S., Libiger O. (2015). Family income, parental education and brain structure in children and adolescents. Nat. Neurosci..

[3] Mani A., Mullainathan S., Shafir E., Zhao J. (2013). Poverty impedes cognitive function. Science.

[4] Evans G.W., Schamberg M.A. (2009). Childhood poverty, chronic stress, and adult working memory. Proc. Natl. Acad. Sci. USA.

[5] The Philippines Statistics Authority MDG Watch: Philippines’ Progress based on the MDG Indicators. http://psa.gov.ph/sites/default/files/kmcd/MDG%20Watch%20as%20of%20May2016.pdf.

[6] The Philippines Statistics Authority The 2015 Census of Population and Housing. https://www.psa.gov.ph/content/highlights-philippine-population-2015-census-population.

[7] The Philippines Statistics Authority (2016). Philippines in Figures: 2016.

[8] Alba M. (1981). Estimating Literacy Rate: A Study Relating Literacy Rate With Combined Gross Elementary and Secondary Schools Enrolment Rate. Philipp. J. Dev..

[9] Brooker S., Okello G., Njagi K., Dubeck M.M., Halliday K.E., Inyega H., Jukes M.C. (2010). Improving educational achievement and anaemia of school children: Design of a cluster randomised trial of school-based malaria prevention and enhanced literacy instruction in Kenya. Trials.

[10] The Philippines National Statistics Office Assessment of Indicators. The Philippines Millennium Development Goals. http://nap.psa.gov.ph/stats/mdg/assessment.asp.

[11] Ericta N.C., Collado G.M.P., The Philippines National Statistics Office (2008). 2008 FLEMMS Final Report.

[12] Leffard S.A., Miller J.A., Bernstein J., DeMann J.J., Mangis H.A., McCoy E.L. (2006). Substantive validity of working memory measures in major cognitive functioning test batteries for children. Appl. Neuropsychol..

[13] Foster C., Duran-Flores D., Dumars K.W., Stills S. (1985). Screening for developmental disabilities. West. J. Med..

[14] United Nations Educational Scientific and Cultural Organization (2005). Understanding of literacy. Education for All Global Monitoring Report 2006: Literacy for life.

[15] Wolf M.S., Curtis L.M., Wilson E.A., Revelle W., Waite K.R., Smith S.G., Weintraub S., Borosh B., Rapp D.N., Park D.C. (2012). Literacy, cognitive function, and health: Results of the LitCog study. J. Gen. Intern. Med..

[16] Huppert A.F., Gardener E., McWilliam B., Banks J., Breeze E., Lessof C., Nazroo J. (2006). Cognitive function. Retirement, health and relationships of the older population in England: THE 2004 ENGLISH LONGITUDINAL STUDY OF AGEING (Wave 2).

[17] Marques dos Santos L., Neves dos Santos D., Bastos A.C., Assis A.M., Prado M.S., Barreto M.L. (2008). Determinants of early cognitive development: Hierarchical analysis of a longitudinal study. Cad Saude Publica.

[18] Tearne J.E. (2015). Older maternal age and child behavioral and cognitive outcomes: A review of the literature. Fertil. Steril..

[19] Ursache A., Noble K.G. (2016). Neurocognitive development in socioeconomic context: Multiple mechanisms and implications for measuring socioeconomic status. Psychophysiology.

[20] Guernier V., Brennan B., Yakob L., Milinovich G., Clements A.C., Soares Magalhães R.J. (2017). Gut microbiota disturbance during helminth infection: Can it affect cognition and behaviour of children?. BMC Infect. Dis..

[21] Stensgaard A.S., Utzinger J., Vounatsou P., Hurlimann E., Schur N., Saarnak C.F., Simoonga C., Mubita P., Kabatereine N.B., Tchuem Tchuente L.A. (2013). Large-scale determinants of intestinal schistosomiasis and intermediate host snail distribution across Africa: Does climate matter?. Acta Trop..

[22] Soares Magalhães R.J., Clements A.C. (2011). Mapping the risk of anaemia in preschool-age children: The contribution of malnutrition, malaria, and helminth infections in West Africa. PLoS Med..

[23] Soares Magalhães R.J., Salamat M.S., Leonardo L., Gray D.J., Carabin H., Halton K., McManus D.P., Williams G.M., Rivera P., Saniel O. (2014). Geographical distribution of human Schistosoma japonicum infection in The Philippines: Tools to support disease control and further elimination. Int. J. Parasitol..

[24] Soares Magalhães R.J., Clements A.C. (2011). Spatial heterogeneity of haemoglobin concentration in preschool-age children in sub-Saharan Africa. Bull. World Health Organ..

[25] Soares Magalhães R.J., Salamat M.S., Leonardo L., Gray D.J., Carabin H., Halton K., McManus D.P., Williams G.M., Rivera P., Saniel O. (2015). Mapping the Risk of Soil-Transmitted Helminthic Infections in the Philippines. PLoS Negl. Trop. Dis..

[26] Owada K., Nielsen M., Lau C.L., Clements A.C.A., Yakob L., Soares Magalhães R.J., Rollinson D., Stothard R. (2017). Measuring the Effect of Soil-Transmitted Helminth Infections on Cognitive Function in Children: Systematic Review and Critical Appraisal of Evidence. Advances in Parasitology.

[27] Owada K., Lau C.L., Leonardo L., Clements A.C.A., Yakob L., Nielsen M., Carabin H., Soares Magalhaes R.J. (2018). Spatial distribution and populations at risk of A. lumbricoides and T. trichiura co-infections and infection intensity classes: An ecological study. Parasites Vectors.

[28] Hay S.I., Tatem A.J., Graham A.J., Goetz S.J., Rogers D.J. (2006). Global environmental data for mapping infectious disease distribution. Adv. Parasitol..

[29] Environmental Systems Research Institute ArcGIS 10.4 for Desktop. http://www.esri.com/.

[30] Quantum GIS Development Team QGIS Geographic Information System. Open Source Geospatial Foundation Project. https://www.qgis.org.

[31] StataCorp Stata Statistical Software: Release 13. https://www.stata.com.

[32] Assmann S.F., Hosmer D.W., Lemeshow S., Mundt K.A. (1996). Confidence intervals for measures of interaction. Epidemiology.

[33] Soares Magalhães R.J., Clements A.C., Patil A.P., Gething P.W., Brooker S. (2011). The applications of model-based geostatistics in helminth epidemiology and control. Adv. Parasitol..

[34] Levine R.A., Levine S., Schnell-Anzola B., Rowe M.L., Dexter E. (2012). Literacy and Mothering: How Women’s Schooling Changes the Lives of the World’s Children.

[35] Al-Mekhlafi H.M., Mahdy M.A., Sallam A.A., Ariffin W.A., Al-Mekhlafi A.M., Amran A.A., Surin J. (2011). Nutritional and socio-economic determinants of cognitive function and educational achievement of Aboriginal schoolchildren in rural Malaysia. Br. J. Nutr..

[36] Grigorenko E.L., Sternberg R.J., Jukes M., Alcock K., Lambo J., Ngorosho D., Nokes C., Bundy D.A. (2006). Effects of antiparasitic treatment on dynamically and statically tested cognitive skills over time. J. Appl. Dev. Psychol..

[37] Belizario V.Y., Totanes F.I., de Leon W.U., Matias K.M. (2014). School-based control of soil-transmitted helminthiasis in western Visayas, Philippines. Southeast Asian J. Trop. Med. Public Health.

[38] Sustainable Development Goals Fund From MDGs to SDGs. http://www.sdgfund.org/mdgs-sdgs.

[39] Belizario V.Y., Liwanag H.J., Naig J.R., Chua P.L., Madamba M.I., Dahildahil R.O. (2015). Parasitological and nutritional status of school-age and preschool-age children in four villages in Southern Leyte, Philippines: Lessons for monitoring the outcome of Community-Led Total Sanitation. Acta Trop..

[40] Spears D., Ghosh A., Cumming O. (2013). Open defecation and childhood stunting in India: An ecological analysis of new data from 112 districts. PLoS ONE.

[41] United Nations Children’s Fund, World Health Organization (2015). Progress on Sanitation and Drinking Water—2015 Update and MDG Assessment.

[42] Oria R.B., Murray-Kolb L.E., Scharf R.J., Pendergast L.L., Lang D.R., Kolling G.L., Guerrant R.L. (2016). Early-life enteric infections: Relation between chronic systemic inflammation and poor cognition in children. Nutr. Rev..

[43] Brouwer C., Hoevenaars J.P.M., van Bosch B.E., Hatcho N., Heibloem M., International Institute for Land Reclamation and Improvement (ILRI), Food and Agriculture Organization of the United Nations (FAO) Land and Water Development Division (1992). Water sources and water availability. Irrigation Water Management: Training Manual No. 6—Scheme Irrigation Water Needs and Supply.

[44] Food and Agriculture Organization (2000). The Elimination of Food Insecurity in the Horn of Africa.

[45] Sarsour K., Sheridan M., Jutte D., Nuru-Jeter A., Hinshaw S., Boyce W.T. (2011). Family socioeconomic status and child executive functions: The roles of language, home environment, and single parenthood. J. Int. Neuropsychol. Soc..

[46] Dayrit H., Ti H.L., Facon T. (2001). The Philippines: Formulation of a national water vision. From Vision to Action: A Synthesis of Experiences in Southeast Asia.

[47] Chandra A., McNamara E.K., Dargusch P., Caspe M.A., Dalabajan D. (2017). Gendered vulnerabilities of smallholder farmers to climate change in conflict-prone areas: A case study from Mindanao, Philippines. J. Rural Stud..

[48] Brito N.H., Noble K.G. (2014). Socioeconomic status and structural brain development. Front. Neurosci..

[49] The Inter-Agency Regional Analysts Network (2016). Asia Report: Socio-Economy of Chronic Malnutrition in the Philippines: A Preliminary Key Trends Analysis by 2030.

[50] United Nations Children’s Fund (2013). Improving Child Nutrition: The Achievable Imperative for Global Progress.

[51] Kiechle E.S., Bailey S.C., Hedlund L.A., Viera A.J., Sheridan S.L. (2015). Different Measures, Different Outcomes? A Systematic Review of Performance-Based versus Self-Reported Measures of Health Literacy and Numeracy. J. Gen. Intern. Med..

[52] Bradley R.H., Corwyn R.F. (2002). Socioeconomic status and child development. Annu. Rev. Psychol..

[53] Hutcheon J.A., Chiolero A., Hanley J.A. (2010). Random measurement error and regression dilution bias. BMJ.

[54] Bradley R.H., Caldwell B.M. (1979). Home observation for measurement of the environment: A revision of the preschool scale. Am. J. Ment. Defic..

[55] Bradley R.H. (2015). Constructing and Adapting Causal and Formative Measures of Family Settings: The HOME Inventory as Illustration. J. Fam. Theory Rev..

[56] Bradley R.H., Caldwell B.M. (1984). The relation of infants’ home environments to achievement test performance in first grade: A follow-up study. Child Dev..

[57] Bradley R.H. (1993). Children’s home environments, health, behavior, and intervention efforts: A review using the HOME inventory as a marker measure. Genet. Soc. Gen. Psychol. Monogr..

[58] Farah M.J., Betancourt L., Shera D.M., Savage J.H., Giannetta J.M., Brodsky N.L., Malmud E.K., Hurt H. (2008). Environmental stimulation, parental nurturance and cognitive development in humans. Dev. Sci..

[59] Mireku M.O., Boivin M.J., Davidson L.L., Ouedraogo S., Koura G.K., Alao M.J., Massougbodji A., Cot M., Bodeau-Livinec F. (2015). Impact of helminth infection during pregnancy on cognitive and motor functions of one-year-old children. PLoS Negl. Trop. Dis..

[60] Alvarez-Galvez J. (2016). Discovering complex interrelationships between socioeconomic status and health in Europe: A case study applying Bayesian Networks. Soc. Sci. Res..

[61] Lewis F.I., McCormick B.J. (2012). Revealing the complexity of health determinants in resource-poor settings. Am. J. Epidemiol..

[62] Levine R.A., Rowe M.L. (2009). Maternal literacy and child health in less-developed countries: Evidence, processes, and limitations. J. Dev. Behav. Pediatr..

