# Supplementary material for: Determinants of Spatial Heterogeneity of Functional Illiteracy among School-Aged Children in the Philippines: An Ecological Study
Source: Int J Environ Res Public Health. 2019 Jan 7;16(1):137. doi: 10.3390/ijerph16010137 (PMC6339103; doi:10.3390/ijerph16010137)
Supplement: Supplementary file 1 [file ijerph-16-00137-s001.pdf]

Supplementary Materials

Text S1. Sampling methods of the Functional Literacy, Education and Mass Media Survey (FLEMMS)

Functional literacy data using in this study were collected during the 2008 Functional Literacy, Education and Mass Media Survey (FLEMMS). Primary sampling units (PSUs) within a region were stratified based on the proportion of households in the barangay engaged in agricultural activities and per capita income of the city/municipality. In the first stage, PSU were selected with probability proportional to the number of households in the 2000 CPH. PSUs consisted of a barangay or a group of contiguous barangays. In the second stage, in each sample PSU, EAs were selected with probability proportional to the number of households in 2000 CPH. An EA is defined as an area with discernible boundaries consisting of approximately 350 contiguous households. In the third stage, from each sampled EA, housing units were selected using systematic sampling. For operational considerations, at most 30 housing units were selected per sample [1]. All sample households were interviewed from November 20, 2008 to December 13, 2008 [1]. According to the FLEMMS report, the response rate for the 2008 FLEMMS survey was 94.7% and 87.5% for the household survey and the individual survey respectively.

Text S2. The original 2008 FLEMMS functional literacy classifications

According to 2008 FLEMMS [1] functionally literacy are defined as "those who can at least read, write, compute and/or comprehend, this also classifies persons who graduated from high school or completed higher level of education as functional literate". In the original FLEMMS functional literacy levels were stratified into four classifications: 1) those who cannot read and write were classified as functional illiterate; 2) those who can read and write (who can read and write with understanding a simple message in any language or dialect) were considered as basic literate or has simple literacy; 3) those who can read, write and compute were considered as moderate functional literate; and 4) those who can read, write, compute and comprehend (with a significantly higher level of literacy which includes not only reading and writing skills but also numerical and comprehension skills) were considered as functional literate. According to 2008 FLEMMS [1], participants who are classified as high school students were also considered as functional literate.

According to the original 2008 FLEMMS classification [1], participants who were classified as high school students were automatically considered as functionally literate. However, the authors considered that this decision introduced undue ambiguity in the

definition of functional literacy (i.e. being high school students may not necessarily imply that they are functionally literate). For this reason, we excluded those who are classified in this category (i.e. approximately 17% of participants were excluded due to this reason).

Sociodemographic indicators

We used data from the FLEMMS household and individual questionnaires on school-aged children and the heads of households, including age, sex, marital status, education level, employment, and occupation status. Highest education attainment was categorised into three levels (i.e. no grade completed, elementary level, and high school level). Employment status at the time of the survey was categorised into binary variables (yes or no). Primary occupation of the heads of households was categorised into: worked for private household, worked for government, worked with pay on own family-operated farm or business, and worked without pay on own family operated farm or business.

Household education stimuli and cognitive stimulation

A total of 19 items were selected from FLEMMS as the home inventory-proxy items (Table S1). These included questions such as 'Does your family read newspapers?', 'Does your family listen to radio?', 'Is there a personal computer at home?' All selected items were close-ended questions. The items were used to construct cognitive stimulation sub-indices. All of the individual items were translated into dichotomous (yes or no) variables. This is consistent with the scoring format of the Early Adolescent HOME inventory version for children aged 10 to 15 years old which contains 60 items clustered into 7 sub-index: 1) physical environment, 2) learning materials, 3) modelling, 4) instructional activities, 5) regulatory activities, 6) variety of experience, and 7) acceptance and responsivity, using a binary-choice (yes/no) format in scoring items for the HOME [2,3].

Table S1. Household education stimuli score

Cognitive stimulation sub-index (Based on FLEMMS item number)	Yes (1) / No (0)
Is there a TV at home?	
Is there a phone at home?	
Is there a radio at home?	
Is there a CD at home?	
Is there a karaoke machine at home?	
Is there a personal computer at home?	
Does your family read newspapers?	
Does your family read the magazines?	
Does your family read the posters?	
Does your family watch TV?	
Does your family listen to radio?	
Does your family watch movies?	
Does your family use internet?	
Does your family attend meetings of organisations?	
Does your family own a boat?	
Does your family own a tractor?	
Does your family own a car?	
Does your family own a tricycle?	
Does your family own a bicycle?	
Total score (/19)	

Table S2. Results of semivariograms for prevalence of moderate functional literacy

Moderate functional literacy	Observed data	Model 1	Model 2
Luzon			
Partial sill	0.0295	0.0021	0.0027
Nugget	0.0271	0.0084	0.0082
Practical range (km) [a]	0.011 (1.22)	0.061 (6.77)	0.059 (6.55)
% of the variance due to clustering [b]	52.16	20.39	24.81
The Visayas			
Partial sill	0.05	0.0056	0.0053
Nugget	0.04	0.0051	0.0341
Practical range (km) [a]	0.0001 (0.01)	0.104 (11.54)	0.102 (11.32)
% of the variance due to clustering [b]	55.56	52.37	13.42
Mindanao			
Partial sill	0.0224	0.0011	0.0007
Nugget	0.0593	0.0088	0.0091
Practical range (km) [a]	0.282 (31.30)	0.966 (107.23)	0.383 (42.51)
% of the variance due to clustering [b]	27.37	11.41	7.20

Note: [a] Calculation based on practical range multiplied by 111. 1 decimal degree = 111 km, 0.1=11 km, 0.01=1 km, 0.05=5 km, 0.005=500 m; [b] Calculation based on partial sill divided by sill (partial sill + nugget), multiplied by 100

Table S3. Results of semivariograms for prevalence of low functional literacy

Low functional literacy	Observed data	Model 1	Model 2
Luzon			
Partial sill	0.0271	0.0021	0.0005
Nugget	0.0013	0.0084	0.0004
Practical range (km) [a]	0.006 (0.67)	0.061 (6.77)	0.059 (6.55)
% of the variance due to clustering [b]	95.28	20.44	57.47
The Visayas			
Partial sill	0.005	0.0006	0.0006
Nugget	0.001	0	0
Practical range (km) [a]	0.000 (0.01)	0.045 (4.99)	0.045 (4.99)
% of the variance due to clustering [b]	83.33	100.00	100.00
Mindanao			
Partial sill	0.0006	0.0006	0.0006
Nugget	0.0099	0.0004	0.0005
Practical range (km) [a]	1.645 (180.98)	0.449 (49.84)	0.449 (49.84)
% of the variance due to clustering [b]	5.93	63.16	54.55

Notes: [a] Calculation based on practical range multiplied by 111. 1 decimal degree = 111 km, 0.1=11 km, 0.01=1 km, 0.05=5 km, 0.005=500 m; [b] Calculation based on partial sill divided by sill (partial sill + nugget), multiplied by 100

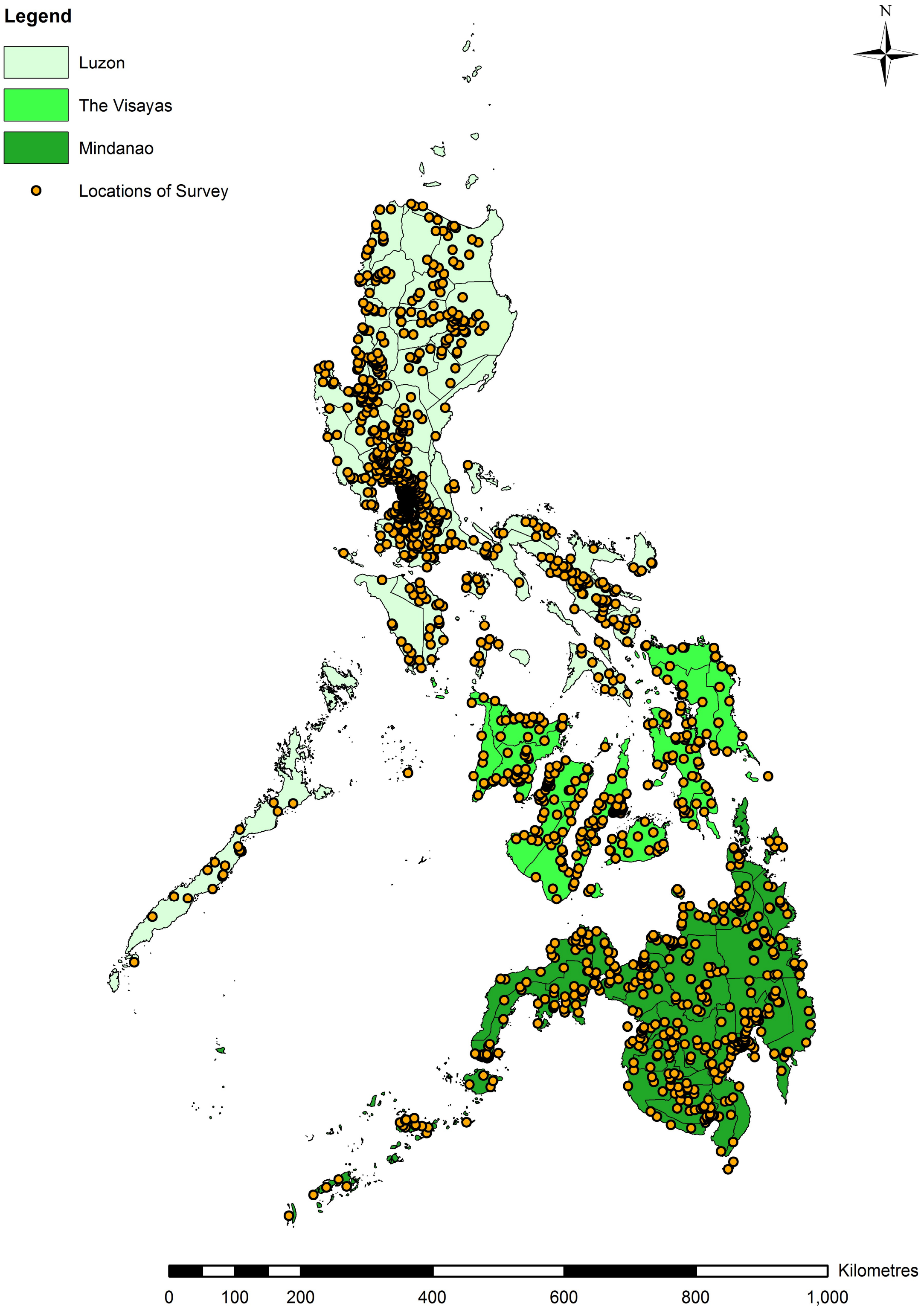

Figure S1. Map of 2008 FLEMMS survey locations

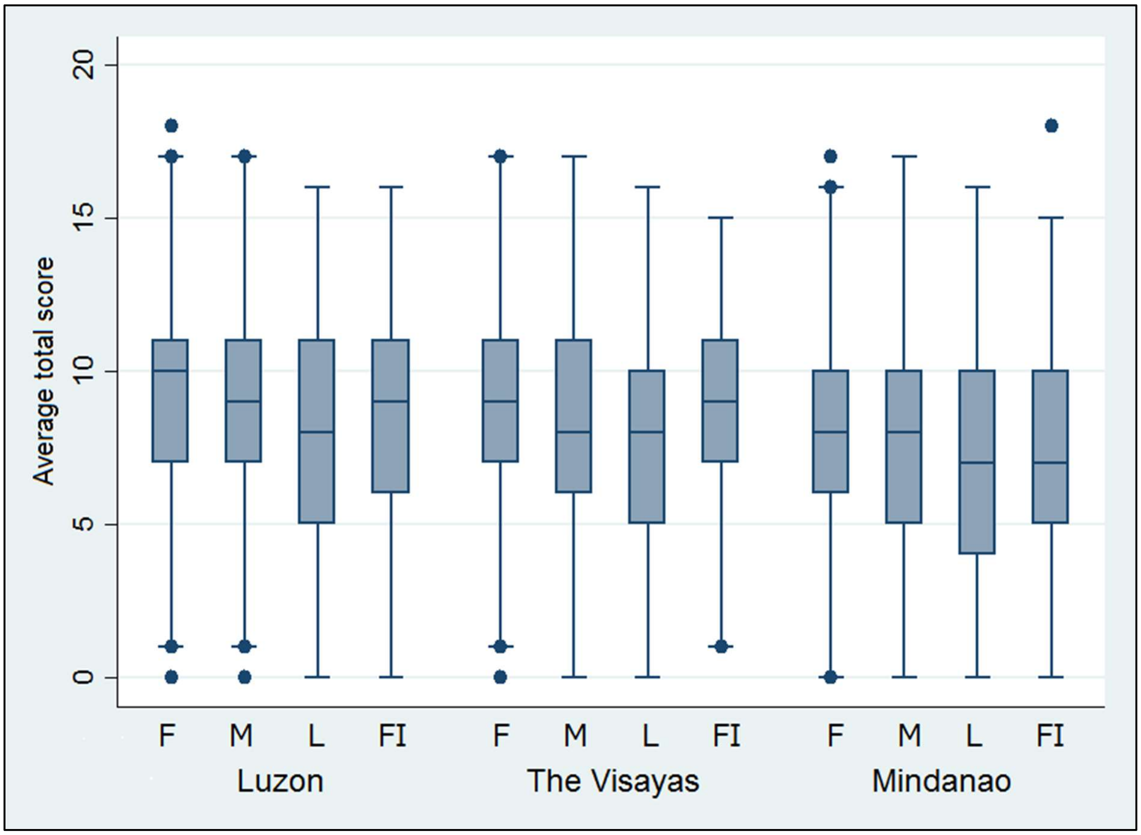

Figure S2. Box plot showing the relationship between household education stimuli average total score and functional literacy indicators, by region

Note: This box plot shows that the household education stimuli average total scores were lower in Mindanao than in Luzon and the Visayas for all literacy levels. F = Functional literacy, M = Moderate functional literacy, L = Low functional literacy, FI = Functional illiteracy. Luzon P-value = 0.000; The Visayas P-value = 0.015; Mindanao P-value = 0.023.

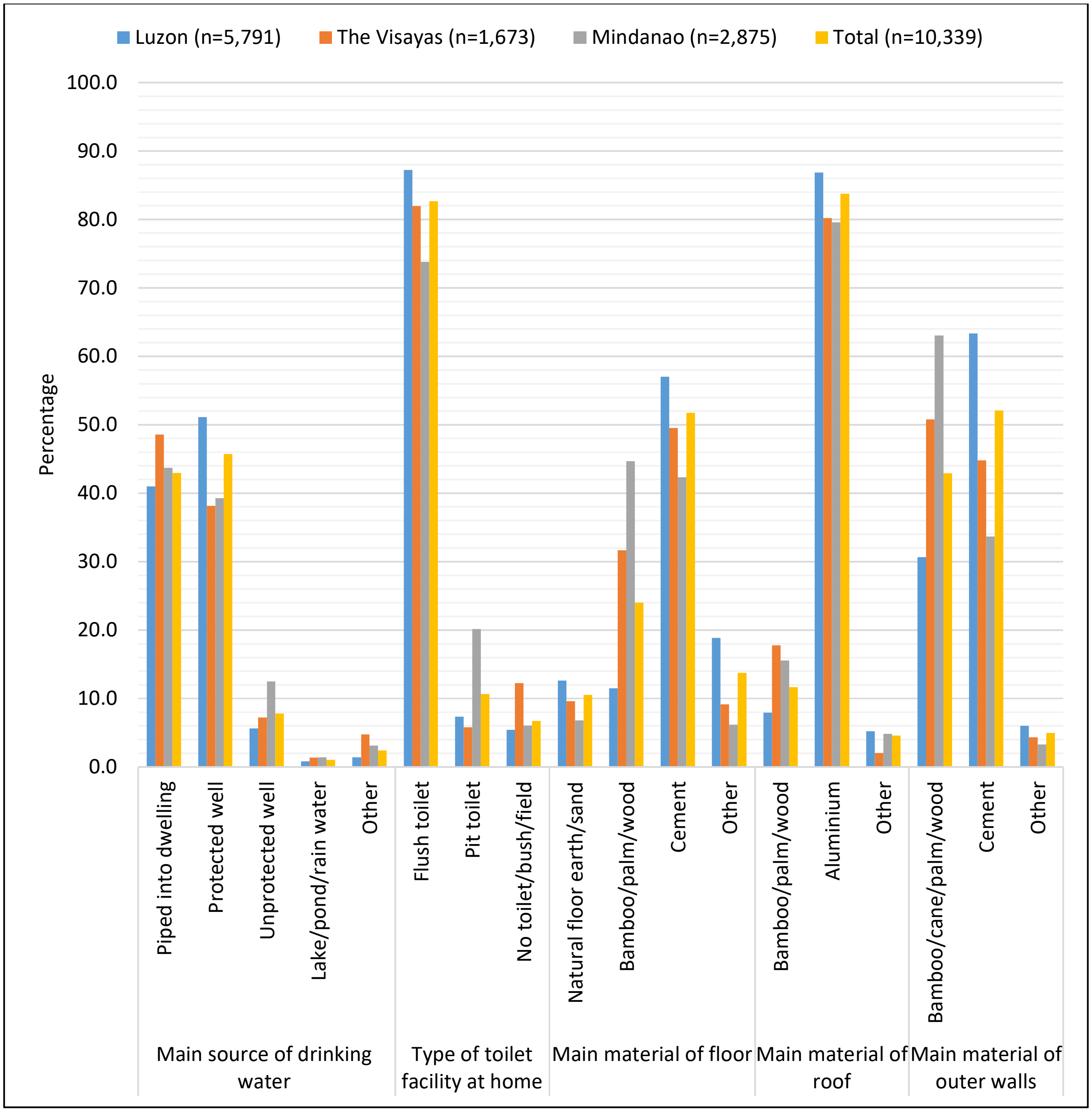

Figure S3. Bar graph showing basic household WASH characteristics

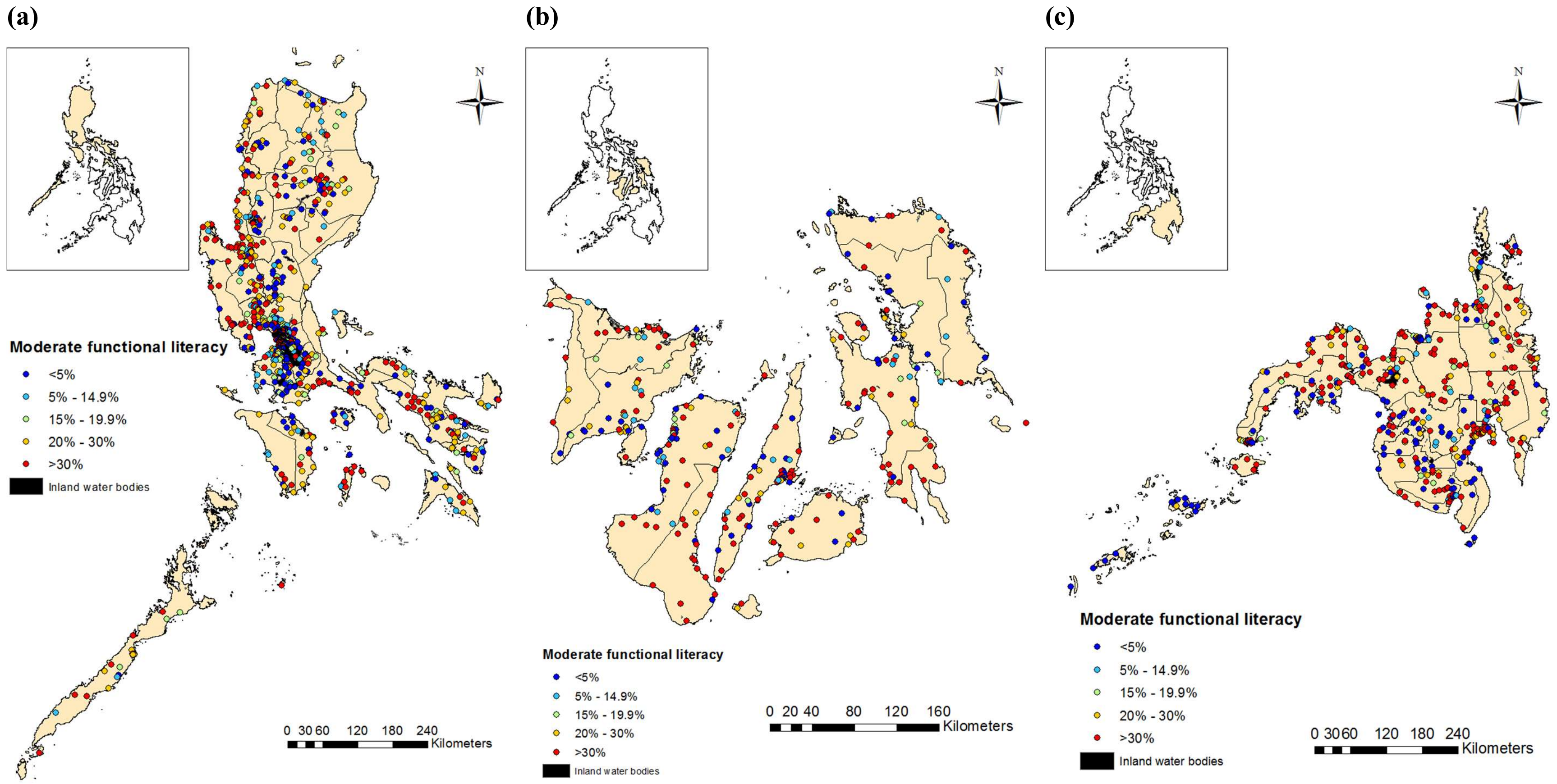

Figure S4. Maps of prevalence of moderate functional literacy of school-aged children in the Philippines by region: **(a)** Luzon; **(b)** the Visayas, and **(c)** Mindanao

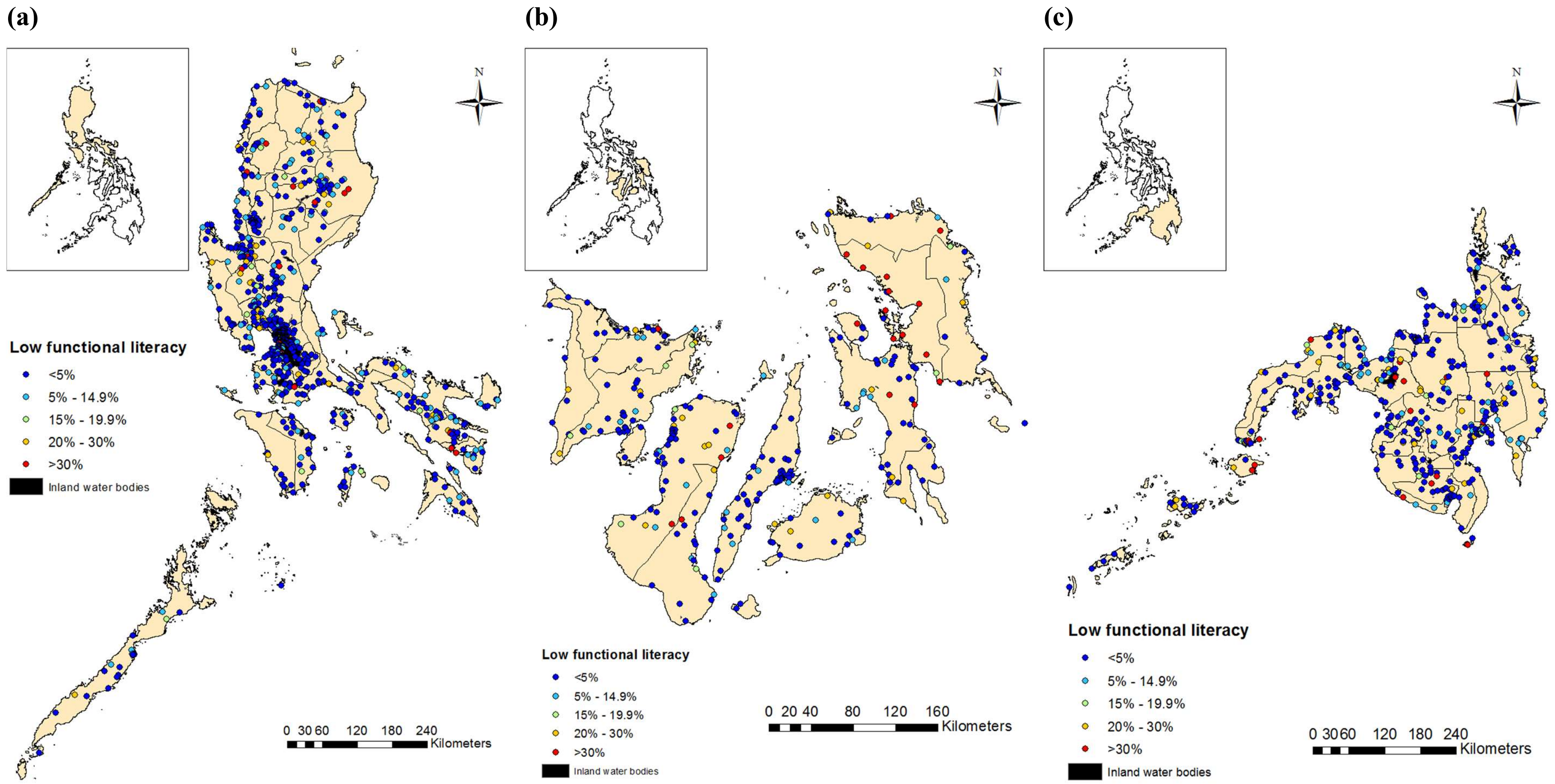

Figure S5. Maps of prevalence of low functional literacy of school-aged children in the Philippines by region: **(a)** Luzon, **(b)** the Visayas, and **(c)** Mindanao

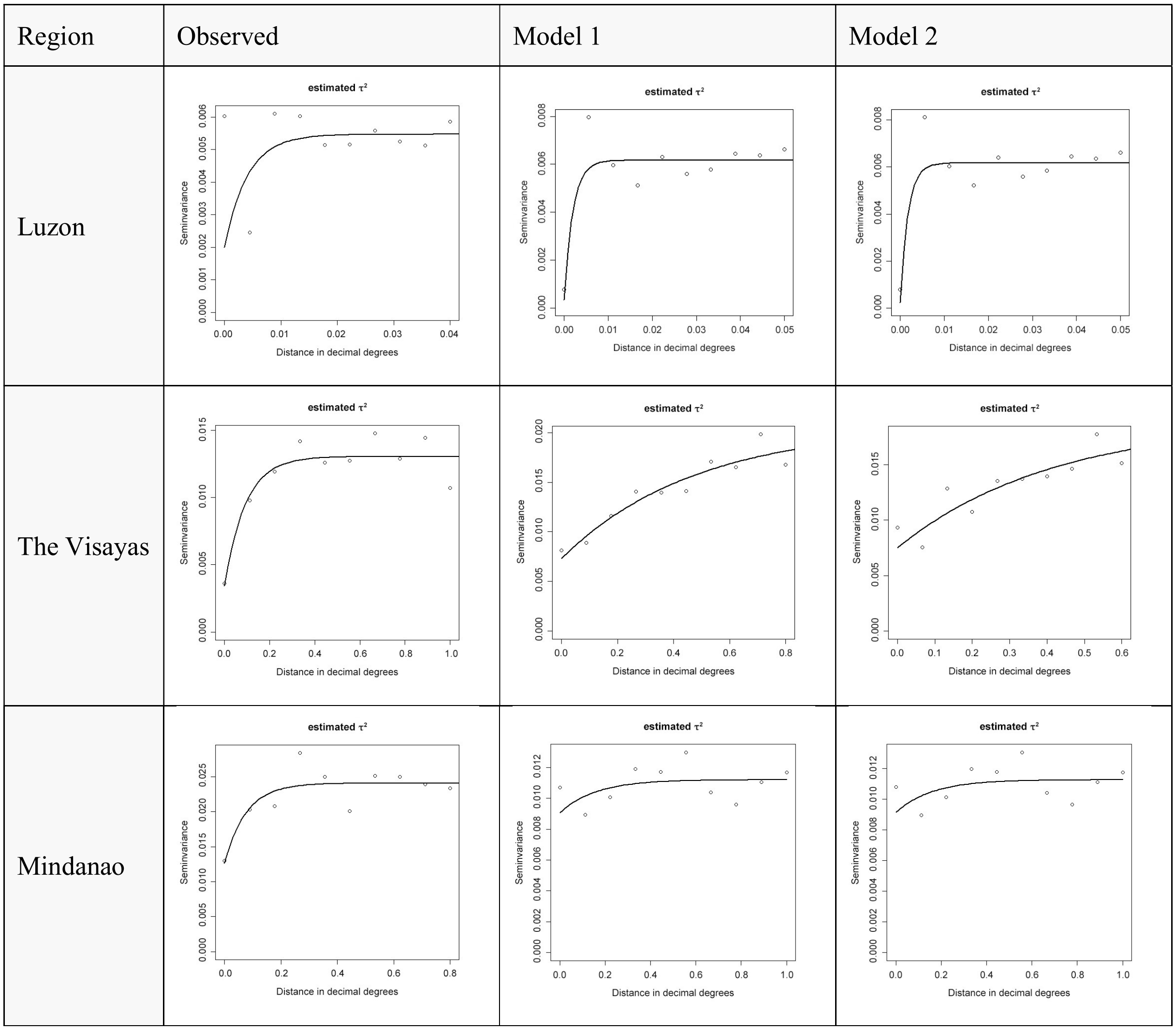

Figure S6. Semivariograms of prevalence of functional illiteracy in school-aged children. Note: Semivariograms of prevalence of observed functional illiteracy indicators and residuals for the final multinomial models (residual semivariograms) at each region to examine the presence of spatial autocorrelation.

Region	Observed	Model 1	Model 2	Model 3
Luzon	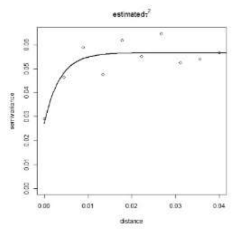	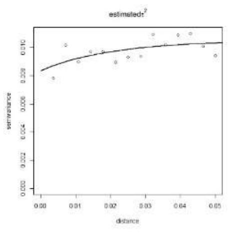	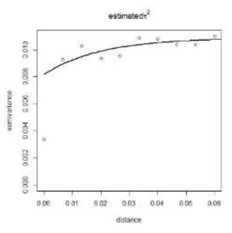	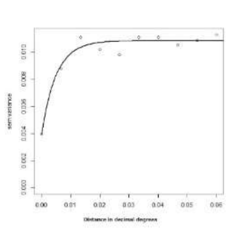
The Visayas	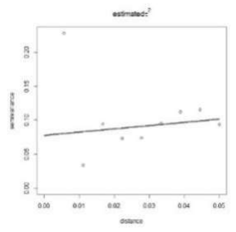	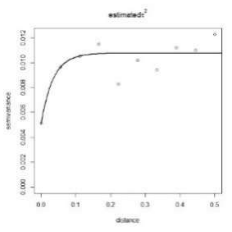	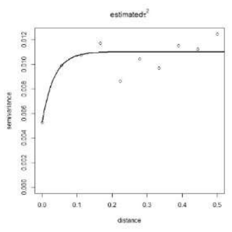	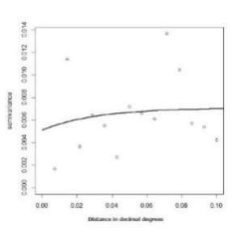
Mindanao	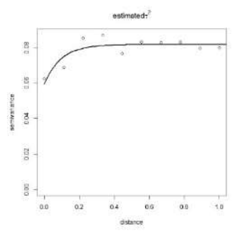	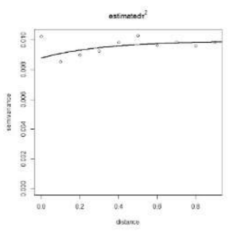	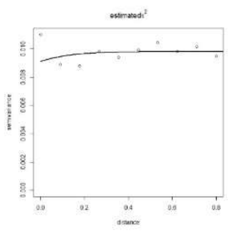	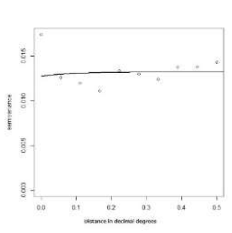

Figure S7. Semivariograms of prevalence of moderate functional literacy in school-aged children. Note: Semivariograms of prevalence of observed moderate functional literacy indicators and residuals for the final multinomial models (residual semivariograms) at each region to examine the presence of spatial autocorrelation.

Region	Observed	Model 1	Model 2	Model 3
Luzon	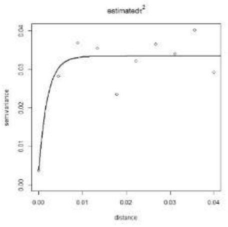	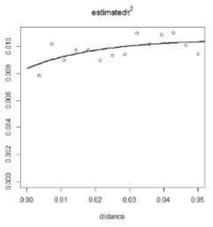	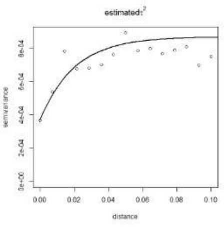	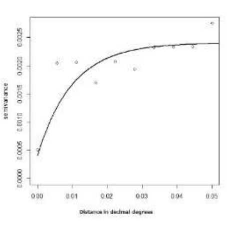
The Visayas	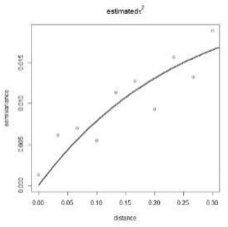	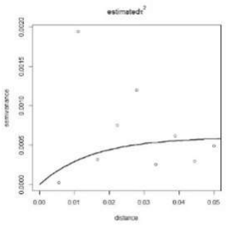	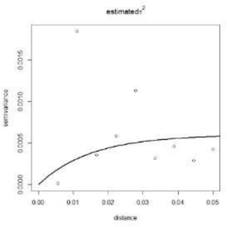	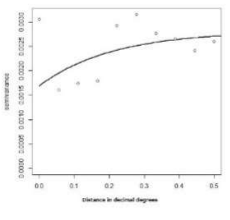
Mindanao	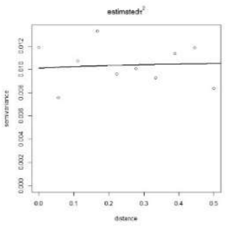	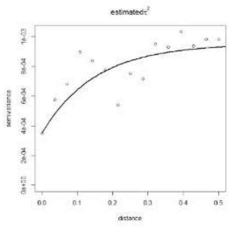	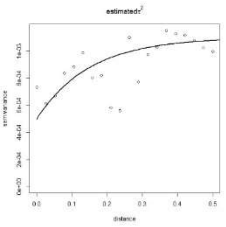	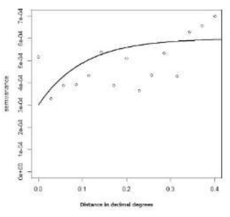

Figure S8. Semivariograms of prevalence of low functional literacy in school-aged children

Note: Semivariograms of prevalence of observed low functional literacy indicators and residuals for the final multinomial models (residual semivariograms) at each region to examine the presence of spatial autocorrelation.

References

1. The Philippines National Statistics Office. 2008 FLEMMS Final Report. Ericta, N.C., Collado, G.M.P., Eds. The Philippines National Statistics Office: Manila, 2008.
2. Bradley, R.H. Constructing and Adapting Causal and Formative Measures of Family Settings: The HOME Inventory as Illustration. *J Fam Theory Rev* **2015**, *7*, 381-414, doi:10.1111/jftr.12108.
3. Bradley, R.H.; Caldwell, B.M. The relation of infants' home environments to achievement test performance in first grade: a follow-up study. *Child Dev* **1984**, *55*, 803-809.
